# Sustainable smart anti-corrosion coating materials derived from vegetable oil derivatives: a review

**DOI:** 10.1039/d2ra07825b

**Published:** 2023-01-27

**Authors:** Poonam Singh, Anuj Rana, Niranjan Karak, Indresh Kumar, Sravendra Rana, Pankaj Kumar

**Affiliations:** a University of Petroleum & Energy Studies (UPES), School of Engineering, Energy Acres Bidholi Dehradun 248007 India srana@ddn.upes.ac.in pkumar@ddn.upes.ac.in; b Department of Microbiology, College of Basic Sciences & Humanities, Chaudhary Charan Singh Haryana Agricultural University Hisar 125004 India; c Department of Chemical Sciences, Tezpur University Napaam 784028 India; d Department of Chemistry, Birla Institute of Technology and Science Pilani 333 031 India

## Abstract

Sustainable development is a critical concern in this fast-paced technological world. Therefore, it is essential to employ renewable resources to move towards sustainable development goals (SDGs). The polyols attained from renewable resources, including lignin, chitosan, vegetable oils, cellulose, *etc.* and the polymers derived from them have attracted the attention of the majority of researchers, both in academia and industry. The development of bio-based polymers from vegetable oils start emerging with different properties to generate a value-added system. This review will give an impression to readers about how coatings generated from vegetable oils can find a way towards better protective properties against corrosion either by using fillers or by using molecular structure modifications in the system, thus covering a range of vegetable oil-based self-healing polymers and their application in anti-corrosion coatings.

## Abstract 2

The article covers coating materials using bio-chemical derived molecules (vegetable oils) and their properties including mechanical, self-healing and anti-corrosion properties.

## Graphical abstract

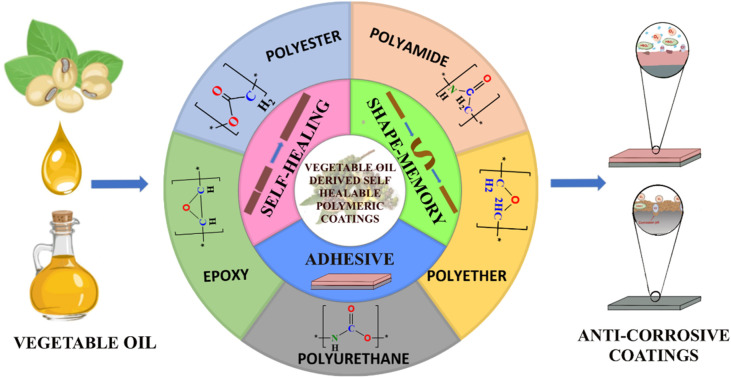

## 1. Introduction

Since mid of the 1900s, petroleum-based polymers have been widely used in the coating industry; however, from the environmental point of view, it is high time we shift towards renewable resources.^1–3^ The major challenge in today's emerging world is the replacement of conventional petroleum-based polymers with renewable polymers, where vegetable oils (VOs) are potential starting materials among other bio-based renewable substances like cellulose, starch, sugar, *etc.*^4–6^ Vegetable oils are the centre of attention owing to their favourable chemical structure, availability, cost, and unsaturated sites for functional group fabrication, thus advantageous for designing the molecules from scratch.^78^ A variety of vegetable oils, *e.g.* castor oil, soybean oil, linseed oil, cotton seed oil, palm oil, Jatropha oil, olive oil, *etc.*, have been used to synthesize polymeric materials.^9–11^ Different parameters are used to understand the characteristic profile of oils, *e.g*. acid value, presence of free fatty acids, saponification value, rancidity, iodine value, *etc.*Table 1 covers the density, saponification value, fatty acid contents and Iodine value of a few commonly used oils.^12–18^ Regarding the synthesis of polymeric materials from natural oil sources, polyols are the mainly used raw substances. To convert vegetable oils into polyols, chemical conversion of oils *via* epoxidation, hydroformylation, ozonolysis, transesterification,^19^ amidation and thiol–ene reaction is performed.^20–22^ Among different vegetable oil-based polymers, polyurethanes are widely used due to their excellent physio-chemical properties.^23^ These properties vary with sources of polyols, preparation methods and functional groups present in resultant polymers.^24,25^

**Table 1:** Density, iodine value, saponification value and molecular structure of some commonly used vegetable oils

S.·No.	Oil name	Fatty acids present	Density (g ml^−1^)	Iodine value (g of *I*_2_/100 g)	Saponification value (mg KOH g^−1^)	Molecular formula
1	Castor oil	Ricinoleic acid, oleic acid, stearic acid, palmitic acid, linoleic acid, linolenic acid	0.96–0.97	102.02	176–186	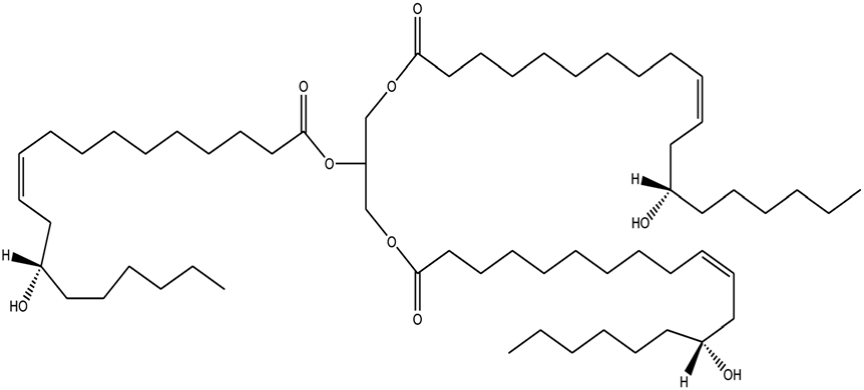
2	Soybean oil	Palmitic acid, stearic acid, oleic acid, linoleic acid, and linolenic acid	—	128.7	—	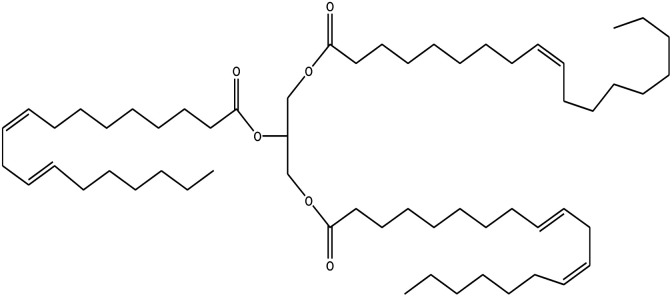
3	Cottonseed oil	Linoleic acid, palmitic acid, oleic acids, stearic acid, myristic acid, palmitoleic acid, linolenic acid	0.918–0.926	109.4	—	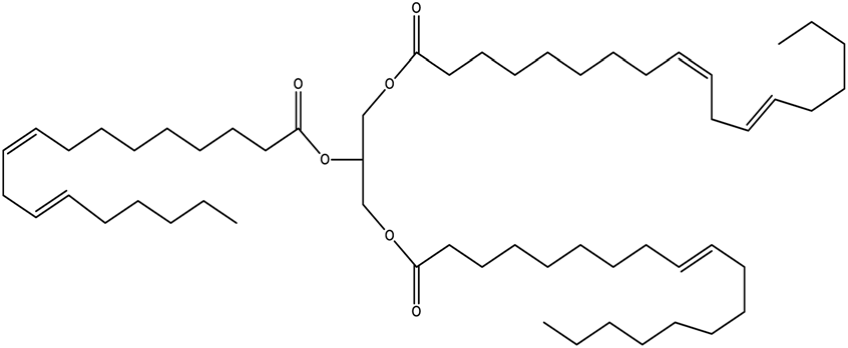
4	Sunflower oil	Palmitic acid, stearic acid, oleic acid, linoleic acid, arachidic acid, linolenic acid, behenic acids	0.897	120	191.7	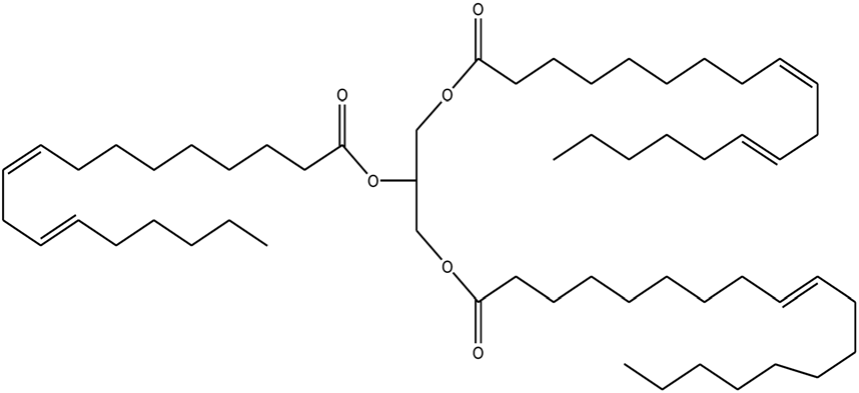
5	Linseed oil	Palmitic acid, stearic acid, linolenic acid, oleic acid	0.93–0.94	180	—	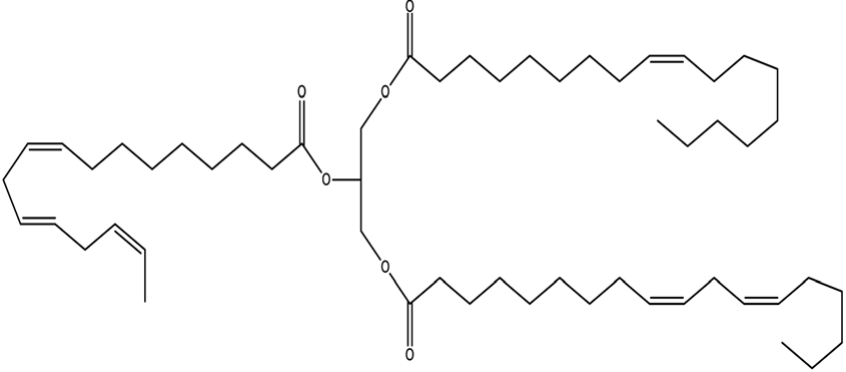
6	Palm oil	Palmitic acid, stearic acid, myristic, oleic acid, linoleic acid, and linolenic acid	—	43.3	—	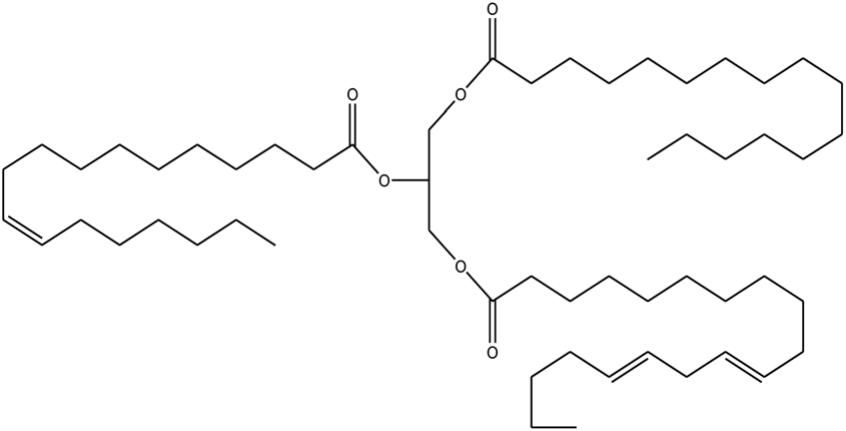
7	Corn oil	Palmitic acid, stearic acid, oleic acid, trans elaidic acid, linoleic acid, linolenic acid, arachidic acid, linoleic acid	0.92–0.93	123.05	—	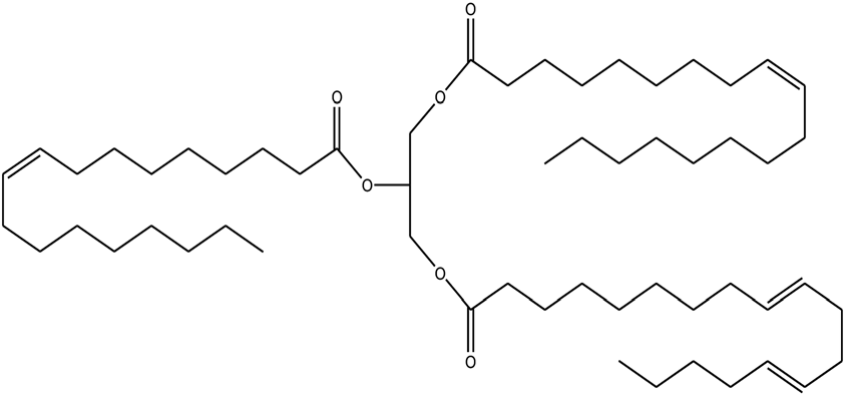
8	Coconut oil	Caprylic acid, capric acid, lauric acid, myristic acid, palmitic acid, stearic acid, oleic acid and linoleic acid		15.1	—	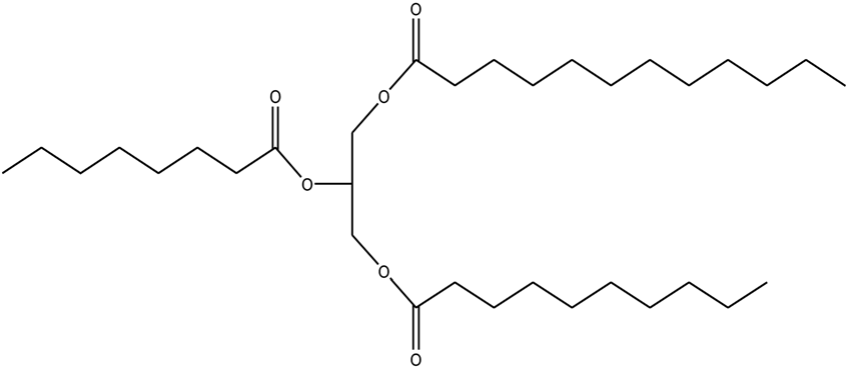
9	Canola oil	α-linolenic acid (omega-3), oleic acid, low amount of saturated acid	0.91–0.92	132	—	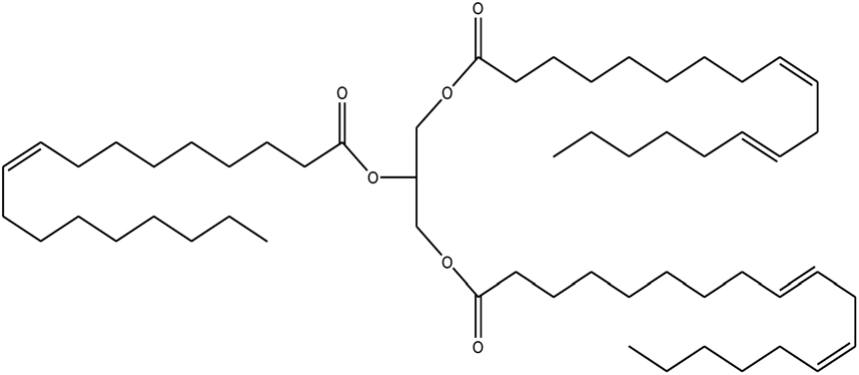
10	Olive oil	Myristic acid, palmitic acid, palmitoleic acid, heptadecanoic acid, heptadecenoic acid, stearic acid, oleic acid, linoleic acid, α-linolenic, arachidic acid, eicosenoic acid, docosanoic acid, lignoceric acid	0.91–0.92	79–91	190–195	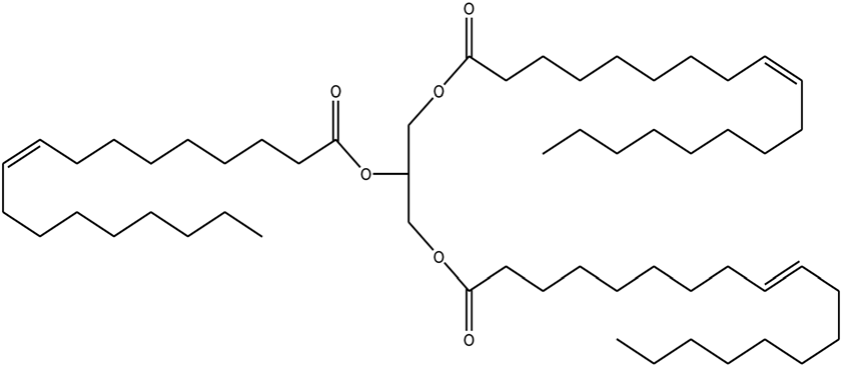
11	Jatropha oil	Oleic acid, linoleic acid, palmitic acid and stearic acid	0.90317	103.6	193.55	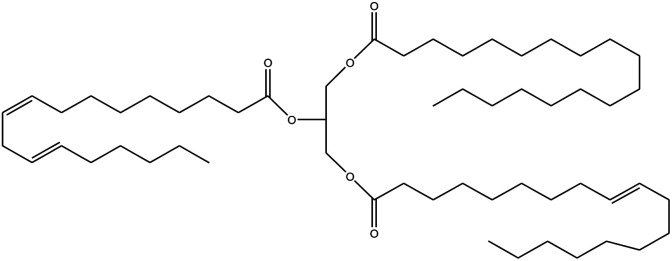

To enhance the durability of polymeric materials, the addition of a self-healing function is highly advantageous.^25–27^ Self-healing can be generally classified into two main groups: intrinsic and extrinsic self-healing materials. These materials primarily differ in their mechanisms as well as their chemistries. While intrinsic self-healing materials are based on supramolecular hydrogen bonding interactions and reversible reactions.^28–32^ Extrinsic self-healing materials require the embedding of microcapsules filled with healing agents in a matrix system or vascular networks.^33–36^ The capsule-based self-healing system (an approach that releases the confined healing agent through the rupture of the microcapsule), and microvascular networks are the two pathways frequently used in the preparation of extrinsic self-healing materials.^22,37–45^ For the addition of self-healing properties, the presence of phenolic groups is advantageous in order to support the cross-linking, which results in a fast self-damage curation^9^ therefore, some phenolic biomolecules known for their affinity towards the modification of vegetable oil-based polymers for coating applications. The –OH (hydroxyl group) containing molecules like flavones, tannin, and flavonoids also act as a suitable corrosion inhibitors. Taking Tannin biomolecules for this purpose provides adequate corrosion protection and self-healing abilities. Tannins are one of the components found in natural extracts and organic products.^46,47^ However, polymeric coatings derived from vegetable oils perform poor chemical and thermal resistance as compared to petroleum-based polymeric coating.^48^ To enhance the thermal and mechanical properties including preventing the moisture/water penetration, a wide range of nanofillers have been incorporated into the coating matrix.^5,13,49–51^ Carbonized materials such as carbon nanotubes (CNTs), graphene, and mesoporous carbon structures have been attracted to researchers due to their chemical, thermal, electrical, and mechanical properties, thus, leading towards the enhancement of mechanical, anti-corrosion, antimicrobial properties.^10,46,52–56^ Carbonaceous hybrid nanostructure materials implanted with different functionalities enhance the self-healing properties. Hydrogen bond donor-acceptor moieties bonded to filler walls show repetitive dynamic repair on damages due to hydrogen bond exchange in the polymeric network *via* click chemistry^30,35,57,58^ vegetable oils derived metals containing polymeric coatings are considered to enhance anti-corrosion and anti-fungal properties, whereas aliphatic tri-ester moiety of oils provides hydrophobic nature and inherent flexibility to the coating.^59–61^ This review will give an impression to readers about how coatings generated from vegetable oils can find a way towards better protective properties against corrosion.

### 1.1. Significance and background

Although so many polymeric protective coatings are present in the market, approx. 3.6% of total GDP (total gross domestic product) is being spent on the maintenance of structural substrates and instruments caused by distruction due to corrosion.^62,63^ Therefore, an intelligent coating able to repair itself and protect the assets from corrosion and improve durability is highly desirable. The whole idea of self-healing coating was inspired by nature, given how natural healing of wounds and cuts occur in living species. The era of self-healing materials is gradually settling in where the macro/micro-cracks in structural materials can close on their own without any external influence, and the scratches on the designed materials can seal up with a complete restoration. In particular, self-healing coatings are helpful to reduce costs and labor associated with coating maintenance for offshore application, where inspection and maintenance are complex due to the location of the asset. However, due to geopolitical challenges, coating industry need to shift its focus from conventional petroleum-based polymers to bio-chemicals derived polymers. Lined up from the biomaterial to smart protective coatings, vegetable oil is worth to utilize for the synthesis of high-performance material, given that oils provide a wide range of fatty acids like oleic acid, linoleic acid, palmitic acid and stearic acid. Leaving an impression of interest, sustainable polymeric protective coatings have been in prime focus for corrosion inhibition of metallic surfaces and thus explored in this report.^6,42^ Along with the inherent properties of vegetable oils, external materials/groups can be easily coupled due to the presence of different functional moieties in oil, as required for different coating applications.^64–66^

## 2. Polymers derived from different vegetable oils (for coating applications)

### 2.1. Castor oil-based polymers

Castor oil, a readily available organic chemical can be extracted *via* solvent extraction or mechanical pressing. In the case of solvent extraction, the main constituent obtained is ricinoleic acid, which is present at around 90% of the whole oil and comes under the euphorbiaceous group. Other components, including linoleic acid, oleic acid, stearic acid, palmitic acid, *etc.*, are present in trace amounts.^9,67^ The presence of –COOH groups in castor oil facilitates the esterification and amidation, whereas double bonds proceed to hydrogenation and epoxidation. In addition, –OH groups promote acetylation and alkoxylation or dehydration, leading to the formation of a different range of polymers, including polyether, polyurethanes, polyesters, polyamides, *etc.* which is being discussed in detail in Table 2 along with their possible applications.

**Table 2:** Polymer synthesis reaction conditions and applications for Castor oil

S. no.	Monomers	Resultant polymer	Catalyst/chain extender (conditions)	Polymerization technique/crosslinking	Fillers	Properties			References
						Self-healing	Mechanical properties	Anti-corrosion	
1	Castor oil (CO) + phthalic anhydride (PA), boric acid (BA) + toluene-2,4-diisocyanate (TDI)	Polyester and polyurethane	—	Polyaddition-esterification	Boron filled polyurethane	—	Thermal stability with decomposition at 250 °C-in NaCl (2.5% wt loss). In HCl (5.47% wt loss)	Anti-corrosive properties	60
2	Glycerol + castor oil+ 1,4- butanediol (BD) + poly(caprolactone) diol (PCL) + calcium oxide+ 2 + 4/2, 6-toluene diisocyanate (TDI) + *N*,*N*-dimethylacetamide	Hyperbranched polyurethane	1,4- butanediol (BD) as chain extender	A_2_ + B_3_ approach		—	Tensile strength up to 11 MPa, elongation at break up to 791%, scratch hardness up to 5 kg, thermal stability up to 261 °C	—	77
3	Castor oil + 1,4-butanediol (BD) + poly(e-caprolactone) diol + sodium thiosulphate + 2,4/2,6-toluene diisocyanate (TDI)	Polyurethanes	—	*In situ* polymerisation/Polyurethane	Sulfur containing reduced graphene oxide (SRGO)	360 W microwave irradiation for 30–60 s providing healing efficiency 99%	Thermal stability up to 421 °C antimicrobial properties against bacteria and fungus, tensile strength-24.3 MPa, tensile modulus-137.74 MPa, elongation-1456 Mpa & toughness-313 MJ m^−3^	—	69
4	Castor oil + 1,4-butanediol (BD) + poly(e-caprolactone) diol + anhydrite ferric chloride + 2,4/2,6-toluene diisocyanate (TDI)	Polyurethane	—	Polyurethane linkage	Iron oxide containing reduced graphene oxide (IO-RGO)	360 W microwave irradiation for 30–60 s with self-healing efficiency 99%	Tensile strength-28.3 MPa, tensile modulus-37.3 MPa, toughness-121.78 MJ m^−3^, & elongation-1180% with strong adhesion	—	70
5	Castor oil + IPDI (iso-phorone diisocyanate)	Polyurethanes,	Stannous octoate (tin 2ethylhexanoate), zirconium 2-(2-(2-(2-aminoethylamino)ethylamino)ethylamino)-ethylphosphonate)	Urethane [–NH–COO–] linkages	—	—	Tensile strength, hydrophobicity, excellent lap shear strength	Anti-corrosion coating, foams, hybrid materials, adhesives	71
6	Castor oil + 2, 2-bis (hydroxyl methyl propionic acid)+ poly (melamine-co-formaldehyde) isobutylated solution (PMF)+ glycerol	Poly-ester-amide	—	A_2_ + B_3_ polycondensation reaction	TiO_2_ nanoparticle	—	Hydrophobic (contact angle 89° to 107°), improves the scratch hardness from 8 to 12.0 kg, thermal stability 452 °C	Anti-corrosive inhibition with *E*_corr_ = −0.192 V, *I*_corr_ = 9.89 × 10^−7^ A cm^−2^, corrosion rate = 0.00016mpy, phase angle = 77° &Rpore = 3.7 × 10^6^ ohm	72
7	Castor oil + iso-phorone diisocyanate (IPDI) + di-methylol propionic acid (DMBA) + methyl ethyl ketone(MET)	Water borne polyurethane	Dibutyltin dilaurate (DBTDL), TEA to neutralize the COOH groups in the polymer	Urethane linkage	Sodium lignosulfonate nanoparticles	—	Excellent UV absorption, hydrophobic with contact angle 81.88°, thermal stability up to 400 °C, tensile strength increases from 10.40 MPa to 14.97 MPa, young modulus 72.58–203.54 MPa & elongation 117.38–71.02%	—	68
8	Castor seed oil + polymeric 4,4′-methylene diphenyl diisocyanate (PMPI) + trimethyl propanone (TMP) + 4-methyl pentan-2-one	Polyurethane		/Amide cross-linking	Carbon nano-materials from eucalyptus globulus leaves	—	Thermal stability up to 399 °C, contact angle- enhances from 71.6° to 87.5°, tensile strength-34 MPa. Elongation-11%	Corrosion inhibition on mild steel, *E*_corr_ – 12.4 mV, *I*_corr_ − 4.4 × 10^−4^ A cm^−2^, *R*_p_ − 7.02 × 10^4^ K ohm cm^2^ & *R*_cor_r − 5.1 × 10^−6^mpy	10
9	Epoxidized sesame oil + castor oil + peracetic acid + diglycidyl-ether bisphenol(DGEBA) + azo-bis (isobutyronitrile)(AIBN)	Epoxy-acrylate	Triphenylphosphine (TPP) as catalyst, hydroquinone (HQ) as inhibitor	Cured at 120 °C −140 °C	—	—	Thermal stability up to 428 °C, good adhesion with lap shear strength-6.39 MPa & T-peel strength 5.78N/25 mm	—	78
10	Castor oil monoglyceride + phthalic anhydride (PA) + maleic anhydride + succinic acid and propylene glycol	Polyester	Initiator-methyl ethyl ketone peroxide. Accelerator- cobalt octoate catalyst- gamma-alumina and formic acid	Two-stage poly-esterification, using dean and Stark type condensation/alkyd chain cross-linking	—	—	Highly thermal stable up to 525 °C	—	79
11	Castor oil + tolulene 2,4-diisocyanate(TDI) + 12-hydroxy-cis-9-octadecenoic acid & diethanol amine(DEA)	Polyurethane	Diethyl ether + dimethyl ketone	Esterification	—	—	Scratch hardness-80 kg, impact resistance −200 lb per inch	Corrosion inhibition efficiency 94.02% & 90.1% in acid and alkaline medium	73
12	Crambe oil + castor oil + trimethyl propane + phthalic anhydride(PA) + butylated hydroxyl toluene(BHT) + lithium hydroxide + *n*-butyl acetate + hemamethylene diidocyante(HDI)	Polyurethanes	Stannous octoate as catalyst	Transesterification	Tannin modification	—	Phase angle = 85°, average thickness of coating-616micro meter	Corrosion inhibition with impedance magnitude from 1.0×10^11^ ohm cm^2^ to 4.1 × 10^10^ ohm cm^2^	46
13	Monomers discussed-10-hydroxy-9-methoxyoctadecanoyl azide/9-hydroxy-10-methoxyoctadecanoyl azide (HMODAz), methyl-N-11-hydroxy9-cis-heptadecen carbamate (MHHDC), and 12-hydroxy-9-cisoctadecenoyl azide (HODEAz)	Polyurethane, polyester, polyamides, epoxy	*N*-methyl diethanolamine (MDEA) and DBTDL, enzymatic catalyst. For polyamide-ruthenium-alkylidene catalyst	Transesterification. For polyester ring opening polymerisation./Urethane linkages, targeted polyester, polyamide, polyurethane, amide linkage. Polycondensation	Montmorillonite, bioactive fillers, CO-based PU/cellulose (CNCs) nanocomposites. CNT, graphene, carbon black, and nanocellulose	Shape-memory thermoplastic pus	Hygroscopic nature, antibacterial activity, tensile strength, hardness, mechanical strength and elastic elongation. Contact angle = 89–107°	Anti-corrosive properties	9
14	Fatty amides of castor oil + pyromellitic dianhydride + alkyd resin + diisocyanates [isophorone diisocyanates (IPDI) + methylene diphenyl diisocyanate (MDI)], and 2,2-bis(hydroxymethyl) propanoic acid	Alkyd resin	Sodium methoxide as catalyst, di-butylene dilaurate (DBTDL)	Amide linkage	Carbon nano fillers	—	Impact resistance = 74.3 lb per inch & 94.5 lb per inch, scratch resistance 05 & 1.5 kg thermal stability up to 356 °C & 370 °C (IPDI & MDI respectively)	Anti-corrosion efficiency 99.6% & 99% on mild steel and wood panels	74
15	l-lysine ethyl ester diisocyanate(LDI), penta-methylate diisocyante(PDI) + hexa-methylene diisocyante + castor oil + 1,3,5-trioxane	Polyurethanes	Silicon based or clay/catalyst -tri-*n*-butylphosphene, chain extender-1,3-propane diol	One-pot synthesis, LDI- two-step esterification method cross-linking density −1.4 MPa	Silicon based clay	Self-curing behaviour	Visco-elastic behaviour, thermally stable up to 290 °C	—	67
16	Castor oil+ 4,4′-disulfanediyldiphenol (DTDP) + (2-(4-aminophenyl)-1,3,2-dioxaborolan-4-yl) methanethiol(SBN) + polyols and 2,2′-disulfanediyldianiline (DTDA) or 4,4′-disulfanediyldianiline (para DTDA)	Polyurethane	Dibutyltin dilaurate (DBTDL)/78 °C	Dynamic disulfide bonds	2,2′-disulfanediyldianiline(DTDA)	Good shape memory and covalent adaptable networks with Rf over 100%	Tensile strength = 38.4 MPa, elongation break- 446%, toughness-33.4 MJ m^−3^, young's modulus-628.3 MPa, contact angle-74.6°–86.3°. After reprocessibility = elongation-190%, tensile strength-15.1 MPa	—	25
17	Polymeric 4,4-methylene diphenyl diisocyanate + 4-methyl-2-pentanone + glycerol + Epichlorohydrin(ECH) + polyepichloro-hydrine PECH-triol	Polyurethanes	PMDI, MIBK	Polycondensation	—	—	Tensile strength = 16.3 MPa, elongation-78% toughness-589 MJ m^−3^. Antimicrobial properties on escherichia coli, pseudomonas aeruginosa, staphylococcus aureus, bacillus subtilis, aspergillusniger, yeast and thermal stability up to 470 °C	Anti-corrosive properties with *E*_corr_ = −93.3 mV, *I*_corr_ = 2.2 × 10^3^ micro A cm^−2^, *R*_p_ = −2.5 × 10^−5^ mm per year	80
18	Castor oil + 2,2-bis-(hydroxymethyl)propionic acid (DMPA) + *P*-toluene-sulfonic acid(*P*-TSA) + mono-hydroxyl-terminated poly(dimethyl-siloxane) PDMSOH	Polyurethane	Hexamethylene diisocyanate trimer (HDIT) as curing agent, dibutyltin dilaurate (DBTDL) as catalyst	Transesterification/urethane linkage	PDMS-OH	Self-cleaning ability	Hydrophobicity with contact angle = 103.8°	—	75
19	Castor oil + dibutyltin dilaurate(DBTDL) + isophorone diisocyanate (IPDI) + triethylamine (Et3N) + dimethylolbutanoic acid (DMBA) + 2aminophenyl disulphide	Water borne polyurethane	—	Di-sulfide linkage	Ti_3_AlC_2_	Self-healable at 60 °C and electromagnetic shielding 99.9%	Tensile strength = 15.74 MPa, elongation = 28.35%, young's modulus = 611.35 MPa, toughness = 4.09 to 19.42 MPa	—	76
20	Epoxidized castor oil + Vanillin + furfuryl amine + paraformaldehyde	Benzoxazine resin/epoxy	180 °C for 2 h, bio-based benzoxazine resin	Crosslinking network	Graphene nano particles (GNP)	Shape memory efficiency = 99% in 19 s, shape fixing efficiency 92%	Thermal stability up to 307 °C, *T*_g_ = 84 °C, strong modulus at100 °C, high vis-NIR absorption	—	81

**Fig. 1:**
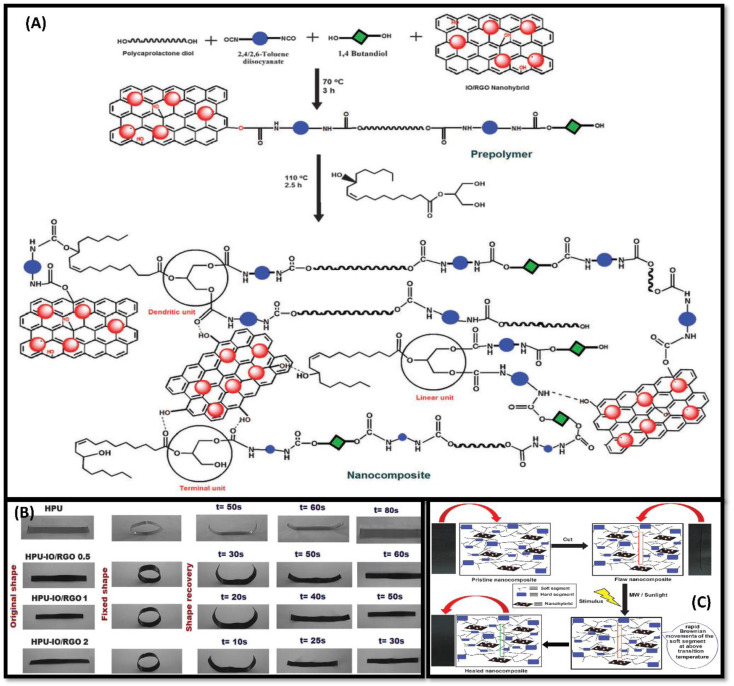
(A) Pictorial representation of synthesis of Nanocomposite HPU-IO-RGO. (B) Representation of nanocomposite showing shape-memory behaviour under MW stimulus. (C) possible depiction for healing mechanism of nano-composite. This figure has been adapted from ref. 70 with permission from Royal Society Of Chemistry, copyright 2015.

To enhance coating properties, filler incorporation is highly advantageous.^68^ Deewan and coworkers studied the effects of incorporating boron-based nanofillers in polyester and polyurethane matrix during synthesis.^60^ The reaction involves esterification of hydroxyl group of castor oil (BCPE) followed by polyaddition between BCPE and Toluene,2,4-diisocyanate (TDI). The coating results in highly effective anti-corrosion properties with no weight loss in the basic medium for 50 hours, whereas a loss in weight was observed in the acidic medium. Good thermal stability (∼220 °C) and physio-chemical properties were observed for boron-polyurethane-based coatings. To understand the significance of castor oil-based shape memory PUs, Karak and coworkers discussed the advantages of using sulfur containing reduced graphene oxide based coatings (SRGO).^69^ The presence of functional groups on the graphene surface gives better dispersion properties in polymeric matrix than to pristine graphene nanosheet. In addition, poly(ε-caprolactone)-diol (PCL) and SRGO were mixed to get the final hyperbranched PU/SRGO (HPU/SRGO) hybrid. Due to the strong interaction between pre-polymer and functionalised GO, improvement in thermal stability and mechanical properties was observed. The presence of PCL helped to achieve excellent shape recovery and shape-healing properties (due to crystalline character) under sunlight and microwave. In addition, authors have studied microwave radiation-promoted SH composites.^70^ The microwave radiation oscillates dipoles, thus helpful in transferring the energy into the polymer matrix. This absorbed energy is beneficial to raise the matrix temperature above *T*_g_ and consequently facilitates the repairing of reported coatings. The synthesis mechanism and exclaimed properties of nanocomposite are substantiated in schematic diagram in [Fig. 1B and C].

Hyperbranched polyurethane iron oxide reduced graphene oxide (HPU-IO-RUO) nano-composites were prepared using different compositions of nanohybrids [Fig. 1A]. The healing efficiency of coating was judged by cutting scratching the surface and calculating the recovery in tensile strength.

Urethane linkages can be obtained *via* reacting isocyanates and alcohols catalysed by tertiary amines (with low steric hindrance to facilitate the reaction), or Pb, Sn based catalysts, *e.g. N*,*N*-dimethyl cyclohexylamine or stannous octoate.^71^ Reacting pre-urethanes along with another polymeric matrix results in the formation of interpenetrating polymer networks (IPN) possessing impressive thermal stability. By controlling the composition of isocyanates and polyols, the matrix demonstrated adhesive properties and is beneficial for vast applications including coatings. Shahidul and coworkers have reported the TiO_2_ dispersed hyperbranched poly-(ester amide)(HBPEA) nanocomposites derived from castor oil using A_2_ + B_3_ approach.^72^ In this process, firstly, *N*,*N*-bis(2-hydroxy ethyl) castor amide (HECA) was synthesized by reacting diethanol amine and sodium methoxide at 110 °C and the addition of castor oil was performed dropwise, which was further used for the synthesis of hyperbranched poly (ester-amide). The reported composite was found to have hydrophobic properties (due to the hydrophobic surface of TiO_2_ increasing cross-linking density) with contact angle in the range of 89° to 107°, and thermal stability of 452 °C.

Moving further, eucalyptus leaves were used to synthesize the carbonized nanoparticles as bio-resourced nano-fillers.^10^ These carbonised nanoparticles were obtained by burning the leaves in atmospheric oxygen and treating them with piranha solution, which leads to delignified carbon nanoparticles. Further, biobased hybrid polyurethane was synthesized by reacting polymeric 4,4′-methylene diphenyl diisocyanate, carbonized nano particles (CNM-COOH) and 4-methyle pentene-2-one along with castor oil. The study found that with increasing the percentage of CNM doping, cross-linking power of urethane and thermal stability (399 °C) was enhanced. The corrosion resistance of the composite coating was improved with *E*_corr_ = 12.4 mV, *I*_corr_ = 4.4 × 10^−4^ A cm^−2^, *R*_p_ = 7.02 × 10^4^ K ohm cm^2^ and *R*_corr_ = 5.1 × 10^−6^ ohm on mild steel.

Kashif and coworkers investigated corrosion inhibition behaviour of castor oil-derived polyurethane, synthesised by reaction of the castor oil with diethanol-amine and sodium methoxide followed by the addition of toluene2,4-diisocyanate(TDI).^73^ The reported polymer matrix possesses high hardness due to TDI (because of aromatic moiety), impact resistance 200 lb per inch, and corrosion inhibition efficiency of 94% & 90% in acidic and alkaline environments, respectively. In another study, it was observed that castor oil-derived alkyd resin has a good affinity towards corrosion inhibition.^74^

**Fig. 2:**
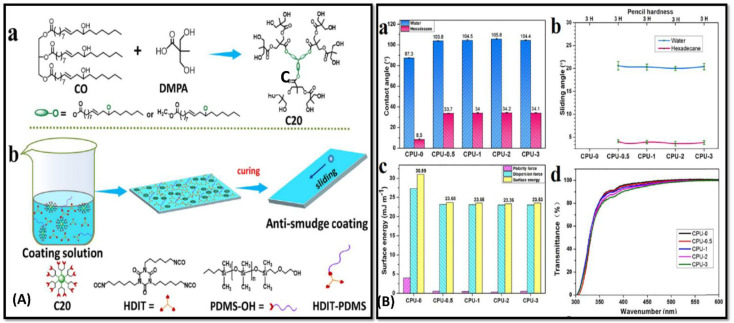
(A) (a) Schematic illustration of synthesis of the Castor Oil-generated hyper branched C20. (b) Diagrammatic demonstration of bio-based ant smudge coating fabrication using HDIT and PDMS-OH (B) (a) comparative graphical study of contact angles on water and hexadecane, (b) graphical representation of pencil hardness and sliding angles of the CPU-0, CPU-0.5, CPU-1, CPU-2, and CPU-3 coatings of water and hexadecane. (d) Optical transmittance and (c) comparative surface energy of the different coatings respectively. This figure has been adapted from ref. 75 with permission from American Chemical Society, copyright 2021.

In order to strengthen the anti-corrosion and hydrophobic properties; lotus leaves inspired self-cleaning coatings is highly advantageous. Wei and coworkers has developed anti-smudge bio-based polymeric coatings ncorporated with poly(dimethyl-siloxane) (PDMS)).^75^ Hyperbranched polyol was synthesised by a transesterification reaction between CO, 2,2-bis (hydroxymethyl)propionic acid (DMPA) and p-toluene-sulfonic acid (*P*-TSA) under nitrogen environment [Fig. 2A(a)]. PDMS-OH was applied to the coating surface to provide the low surface tension and change in contact angle [Fig. 2B]. A study found that it inhibits the deposition of dirt on the surface, and no trace of liquid was left behind. The reported coating was found to repel water, inks, and other organic solvents; thus, it can be used for anti-graffiti and anti-fingerprint purposes [Fig. 2A(b)]. Mechanical and excellent adhesive properties were observed with considerable flexibility and transparent, robust qualities in the coating.

Recently, there has been a high demand for electromagnetic interference (EMI) shielding materials. Jingu Lu and coworkers worked on castor oil-based polyurethanes coating films with properties like self-healing and EMI shielding.^76^ Castor oil was treated with Iso-phorone diisocyanate (IPDI) to synthesize the water-borne polyurethane network. Afterwards, titanium carbide was grafter on the polymer moiety in different proportions along with 2-aminophenyl disulfide (AD). The film prepared was mechanically robust due to presence of H-bonding. Though ADWPU polymeric film synthesised is very effective against electromagnetic waves, after adding Ti_3_AlC_2_ about 99.9% of waves were found to be blocked either by reflecting, scattering or absorbing the radiations.

### 2.2. Soybean oil-based polymers

Soybean oil has majorly been used in the polymeric coating industry for last decade. In spite of high *T*_g_ and strength-based challenges epoxidized soybean oil has remain magnificent starting point for the formation of polymers with diverse properties.^47^ The properties exhibited and possible applications for a different set of polymers derived from soybean oil has been listed in Table 3. Bio-based acrylic monomers show strong hydrophobicity, which is beneficial for the anti-corrosion coating application.^7^ In the wake of exploring soybean oil for synthesising self-healing coating materials, Altuna and coworkers tailored the epoxy resin with gold nanoparticles.^24^ In this study, citric acid was used to synthesise epoxy to synthesise an epoxy-based polymer network followed by the incorporation of gold nanoparticles, where gold NPs were prepared *in situ* through the Turkevich poly(vinyl-pyrrolidone) (PVP) medium. The prepared network is a non-toxic and biologically compatible polymer. The presence of PVP facilitates the linear alignment of the NPs in the polymeric matrix, which promote stress relaxation and self-healing properties of the nanocomposite. The tensile strength of the coating was quite good, and stress relaxation at 60 °C and self-healing properties were well facilitated through localized healing with green laser radiation to ease the reach of thermal effect for the crack healing process. Thiol–ene click reactions are currently in vogue in the self-healing coating industry as it is very economical to carry out at very high-speed kinetics. Prakash and coworkers^83^ used a photo-induced thio-ene click reaction approach to synthesize the castor oil polyol(MCO) and soybean oil(MSO) polyol in the presence of DMPA and ME followed by the formation of thermoplastic PU from MCO and MSO using chain extender 1,4-butanediol (BD) and dibutyltin dilaurate (DBTDL).^24^ The prepared thermoplastic PU was found to have excellent transparency, elasticity and high mechanical strength. Altogether, Kocaman suggested the use of coconut-based bio-fillers to enhance the coating properties.^82^ Acrylated and epoxidized soybean oil (AESO) based epoxy resins can be fused with bio-fillers generated from grinded coconut waste (CW) particles. Coconut waste particles were functionalized with poly(hexafluoro butyl acrylate) (PHFBA) which is hydrophobic in nature. This modification in polymeric matrix benefits the material with several properties like water repellence, high thermal stability, flammability, good acid and alkali resistance which exhibit anti-corrosion properties. The results show that with high cellulosic content, the flammability of the material increases as lignin content is high. Prakash and coworkers suggested that soybean polyols can be prepared through a thio-ene reaction between soybean oil and 2-mercaptoethanol (ME) in the presence of DMPA and THF.^83^ The soybean-based polyol was further used for the formation of polyurethane coating by reacting it with toluene 2,4-diisocyanate (TDI) and DBTDL at room temperature. The resultant coating was found to have good anti-corrosion properties on mild steel panels (tests conducted in 3.5% NaCl and 0.5N HCl aqueous solution). The coating provides excellent tensile strength and barrier properties against corrosion, due to notable high H-bonding in methane linkage between PU coating where no damage or fracture was observed on the coating surface. According to thermal studies, first degradation was observed at 250 °C and second degradation observed at 390 °C due to breakage of ester linkage.

**Table 3:** Soybean oil based bi-polymer synthesis conditions, properties and applications

S. No.	Monomers	Resultant polymer	Catalyst/chain extender (conditions)	Polymerization technique/crosslinking	Fillers	Properties			References
						Self-healing	Mechanical properties	Anti-corrosion	
1	Epoxidized soybean oil (ESO) + citric acid(CA) + hydrogen tetra-chloroaurate(III) + sodium citrate tribasic dihydrate+	Epoxy resin	Polyvinylpyrrolidone (PVP) as stabilising agent	Transesterification	Gold nanoparticles	Self-healing efficiency 100%	Excellent tensile strength 98 Kpa, stress relaxation at 160 °C, with stand load of 100 g (0.98N)	—	24
2	Castor oil + soybean oil + 2,2-dimethoxy-2-phenylacetophenol(DMPA) + 2-mercaptoethol + TDI	Polyurethane	DMPA as photo initiator, dibutyltin dilaurate (DBTDL) as catalyst. 1,4-Butene-diol as chain extender	Photoinduced thiol–ene reactions/urethane linkage thio–ene click reaction	—	—	Tensile strength-13.07 MPa, elongation break- 610% modulus-3.8 MPa, thermal stability up to 450 °C	—	89
3	Bisphenol F-type epoxy resin + acrylated epoxidized soybean oil (AESO) + 2,4,6-tris(dimethylaminomethyl)phenol + 2,2,3,4,4,4-hexafluorobutyl acrylate	Acrylated epoxy	Methyl-nadic anhydride(MNA) and m-xylene diamine(MXDA) as hardner	Plasma enhanced chemical vapor deposition (PECVD) polymerization	Bio-filler from coconut waste	—	Fire resistance, tensile strength-89 MPa + elongation break- 0.64–0.8% and thermal stability up to 260 °C	—	82
3	2-Mercaptoethanol + 2,2-dimethoxy-2-phenylacetophenone (DMPA) + heptadecafluorodecyl methacrylate(FMA), Poly(ethylene glycol)methyl ether methacrylate(EOMA), 2,2′(ethylenedioxy)diethanethiol	Polyurethanes	Dibutyltin dilaurate (DBTDL) as catalyst	Photoinduced thiol–ene reactions, UV irradiation-365 nm, polyaddition reaction	Silane based coating, fluorine and (3-mercaptopropyl)trimethoxy silane (MTS) for silane MSO cross-linking	—	Thermal stability up to 390 °C, contact angle-82.6°, cross-cut adhesion at 82.6°	Corrosion efficiency 86.21%, *E*_corr_ = −490.30 mV, *I*_corr_ = 1.032 × 10−^7^A cm−^2^, *R*_p_ = 5.67 × 10^4^ K ohm cm^2^, corrosion rate = 1.53 × 10−^4^ mm per year	83
4	Epoxy soybean oil + phosphorus oxychloride + vanillin + triethylamine + ethyl acetate + diethylenetriamine + toluene + sodium methoxide	Polyimine	—	Aminolysis and ring–opening reactions, covalent benzaldehyde–amine interactions, exchange between the imine bonds and H-bonds	Multi walled-CNT, trivanillinyl phosphate(TVP)	Self-healing properties with efficiency 93.2%	Tensile strength-25.50 MPa, relaxation time-1796 s at 60 °C, bending strength of 25.51 ± 0.27 MPa, ultra-high stretchability, adhesive, conductive, *T*_g_ = 75 °C	—	84
5	AESO + *tert*-Butyl peroxybenzoate + anhydrous magnesium sulphate (MgSO4) + potassium hydroxide + sodium bicarbonate (NaHCO3)	Different polymer	Monomethyl ether hydroquinone- INHIBITOR, + 4-dimethylaminopyridine (DMAP) as catalyst	Radical polymerization/MVA as the cross-linking agent	—	—	Higher cross-linking density degree, thermal stability up to 455 °C, flexural strength around-2000 MPa	—	90
6	(3-Aminopropyl) triethoxysilane + epoxidized soybean oil(ESO) + 3-mercaptopropyltrimethoxysilane(MPTMS) + 4,4′-methylenebis(phenylisocyante)(MDI)	Polyurethanes	Epoxy ring opening reaction with alkoxy silanes	ZnCl_2_ as initiator, phenyl phosphonic dichloride(PPPC)	—	—	Flame retardancy with LOI value by 26.3%, thermal stability up to 455 °C, burning time up to 30 s to 8 s	—	91
7	Epoxidized soybean oil + *N*,*N*-dimethylacetamide(DNAc) + 4-(dimethylamino)pyridine(DMAP)	Polyhydroxyurethane	Tetrabutylammonium iodide(TBAI) as catalyst	Reversible cyclic carbonate aminolysis and *trans*-carbampylation exchange reactions, thiol-epoxy click chemistry	—	Excellent re-processibility at 110 °C for 40 min	Tensile strength- 0.92 MPa, young's modulus-1.34 MPa, elongation %-233%, gel content-96%	—	92
8	Organo- solve lignin + soybean oil polymer-azide + methacrylic anhydride + epoxidized methacrylated monomer + sodium azide + azobisiso butyronitril(AIBN)	Thermoset elastomers	Thermal Azide−Alkyne cycloaddition click chemistry	4-dimethylaminopyridine(DMAP) as catalyst	Modified lignin	—	Well defined network, excellent elasticity 96−100% after the first cycle, tensile stress-2.12 MPa, tensile strain 134%, strain recovery 96–100%	—	50
9	High oleic soybean oil (HOSO)+ soybean methacrylate monomer(SBMA)	Elastomer latex	Free radical polymerisation	Alumina as initiator, ammonium persulfate (APS) as catalyst	—	Auto oxidative cross-linking	Strain break-359%, young's modulus = 5.4 MPa, stress break-9.7 MPa	High performance coating	7
10	Methacrylated lauric acid(MLA)+ methacrylated oleic acid(MOA) + acylated epoxidized soybean oil (AESO)+ glycidyl methacrylate(GMA)+ lauric acid (LA)+ oleic acid(OA)+	AESO monomer	Catalyst = 2-methylimidazole BPO as initiator	Step growth polymerization and/or oxypolymerization, free radical polymerization	—	Self-healing at 150 °C for 5.5 h	Thermal stability up to 460 °C, tensile strength-3.8 MPa, toughness-0.56 MPa, young's modulus-44.4 MPa, contact angle-80°	—	93
11	Epoxidized soybean oil + 4,4-diaminodiphenyl methane (DDM	Epoxy	4, 4′-Diaminodiphenyldisulfide (APD) as curing agent	di-sulfide exchange reaction	—	Self-healing at 120°	Thermal stability of arounds 370 °C, re-processibility, elongation break-215%, break stress- 3.49 MPa	—	85
12	Glycerol + soybean oil + lead mono-oxide + phthalic anhydride + maleic anhydride	Polyester resins	PbO as catalyst	Ester interchange reaction	—	—	Morphological and physicochemical properties	—	86
13	Acrylated epoxidized SO + glycidyl methacrylate (GMA)+ styrene + oleic acid + lauric acid+	Acrylate epoxy	(2-methylimidazole)-catalyst + benzoyl peroxide(BPO)-initiator + Sn(oct)_2_ as transesterification catalyst	Radical polymerization, transesterification reaction		Self-healing properties	Stress relaxation-22%	—	87
14	Soybean oil + glycidyl methacrylate (GMA)+ hydroquinone monomethyl ether (MEHQ)+ acrylic acid (AA)+ triphenyl phosphine oxide (TPP)+ glycerol + phosphate-buffered saline (PBS)	Acrylate epoxy	MW irradiation	Ring–opening reaction, 2,2′azobis(2-methyl-N-2-hydroxyethyl)propionamide as photo initiator	Methacrylate gelatine (GelMA)	—	Self-stratifying coating, hydrophobic properties, contact angle up to 83.89°	—	88
15	Soyabean oil + poly(methyl methacrylate) (PMMA)+ sodium dodecylsulfate(SDS)	Acrylate epoxy resin	Benzyl dimethyl amine(BDMA) as catalyst	—	Dual microcapsules with diameter 7.8–169.7 mm	Self-healing properties	Thermal stability up to 420 °C	—	94

Generally, imine-based polymers show good mechanical properties and irretrievability, where due to lower in bond energies and easy dissociation, H- bonds provide efficient self-healing properties. Initially, epoxidized soybean oil was treated with trivanillinyl-phosphate (TVP), which was further used to prepare the multilayer-CNT (MWCNT) composites. With the increase in TVP concentration H-bonding abundancy increases while stable imine bonds go down, which results an increase in cross-linking, thus enhances the rigidity of polymer. With the elevation in temperature multi-exchange of network come in existence to achieve self-healing and re-processibility. With a low TVP concentration polymer show rubbery character above *T*_g_, however, a further increase in TVP concentration *T*_g_ increase from 40 °C to 75 °C.^84^

**Fig. 3:**
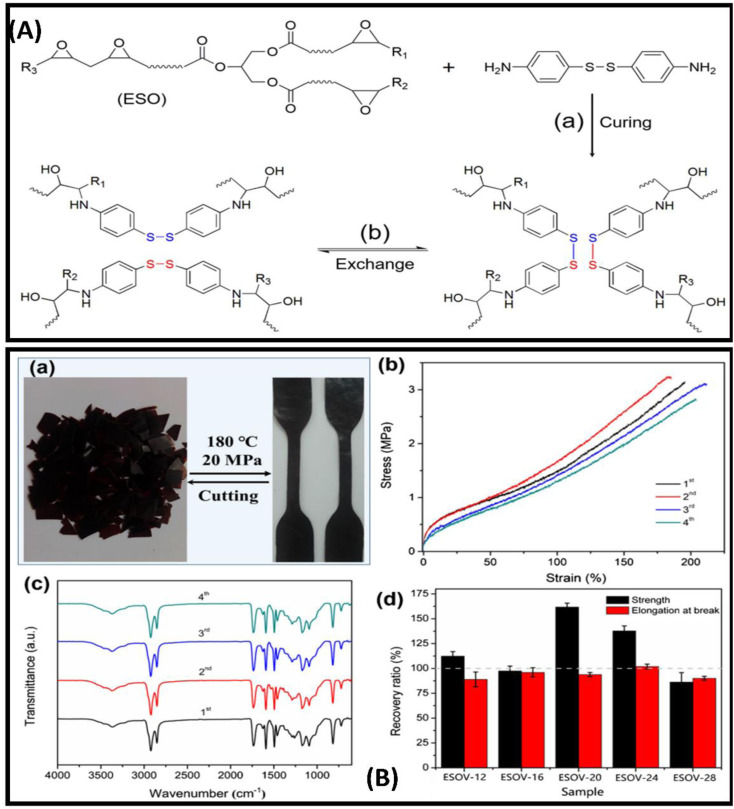
(A) (a) synthesis of ESOV by curing ESO with APD and (b) topological rearrangement of ESOV network *via* disulfide exchange reaction (B) (a)recycled sample of ESOV-28 through cutting and compression molding, (b) stress–strain curves shown and (c) FT-IR spectra of ESOV-28 after each cycle of cutting and compression molding, and (d) recovery ratios of tensile strength and elongation at break of samples after reprocessing for four times. This figure has been adapted from ref. 85 with permission from Elsevier, copyright 2020.

**Fig. 4:**
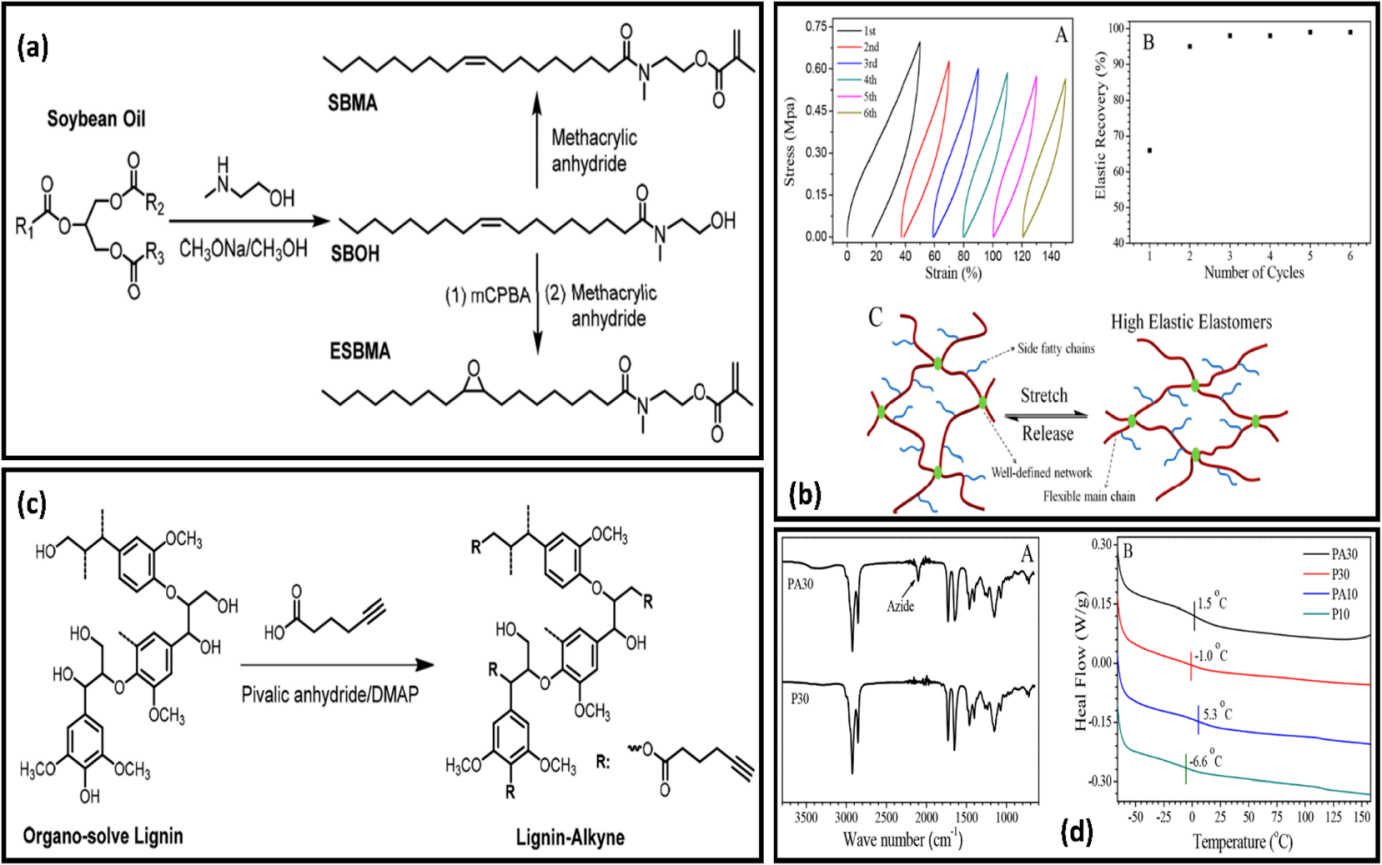
(a) Structural representation of soybean derived Methacrylate Monomers SBMA and ESBMA. (b) (A) Graphical illustration of tensile stress−strain curves of PA30-L10. (B) Elastic recovery with the number of cycles for the PA30-L10. (C) Micro structure model at the stretched and relaxed state of samples. (c) Schematic representation of synthesis of alkyne-functionalized lignin (Lignin-Alkyne) (d) (A) FT-IR spectra of polymers P30, PA30 and DSC curves of copolymers derived from soybean oil (A) and (B) respectively. This figure has been adapted from ref. 50 with permission from American Chemical Society, copyright 2019.

Vitrimeric polymers can be synthesised from the epoxidized oil in the absence of the catalyst. Liu and coworkers developed catalyst-free epoxy based vitrimeric network using soybean oil and 4,4'dithiodiphenylamine(APD) as curing agents [Fig. 3A]. The polymer composite shows excellent tensile strength, reprocesibility bonding strength and super high stretch-ability [Fig. 3B].^85^ Kamal and coworkers produce three different types of novel polyester resins by reacting mono-glyceride (from soybean oil) with phthalic and maleic anhydride. The monoglyceride was obtained by glycerolysis of oil *via* reversible ester interchange reaction.^86^ Yuan and coworkers have reported the synthesis of lignin and soybean oil-based azide-functionalized sustainable elastomers *via* controlled thermal azide−alkyne cycloaddition (TAAC) [Fig. 4a and c] along with the flexibility and appreciable mechanical strength prepared elastomer exhibit excellent elasticity with *T*_g_ less than 5 °C [Fig. 4b and d]^50^. Alongside this, a catalyst-free bio-based vitrimer from soybean oil was done by Guillermina and coworkers. The thermal and mechanical behaviour of the material was found to be controlled by the content of the precursor used.^87^

Despite limited application due to water resistance, methacrylate gelatine can be used in bio-coating to improve moisture resistance. Being able to perform photo-crosslinking reactions and be prepared from renewable resources, methacrylate gelatine is found to enhance hydrophobicity and mechanical properties. Single layer application is reported to be very useful and spontaneously stratifying into layers, whereas multilayer application is quite complex, expensive and energy consuming. Self-stratification is blending incompatible polymers to form a collective multilayer structure. Sahar and coworkers reported that to enhance the mechanical properties and breathability, this self-stratifying light curable AESO-based coating material could be formulated.^88^

### 2.3. Linseed oil-based polymer

**Fig. 5:**
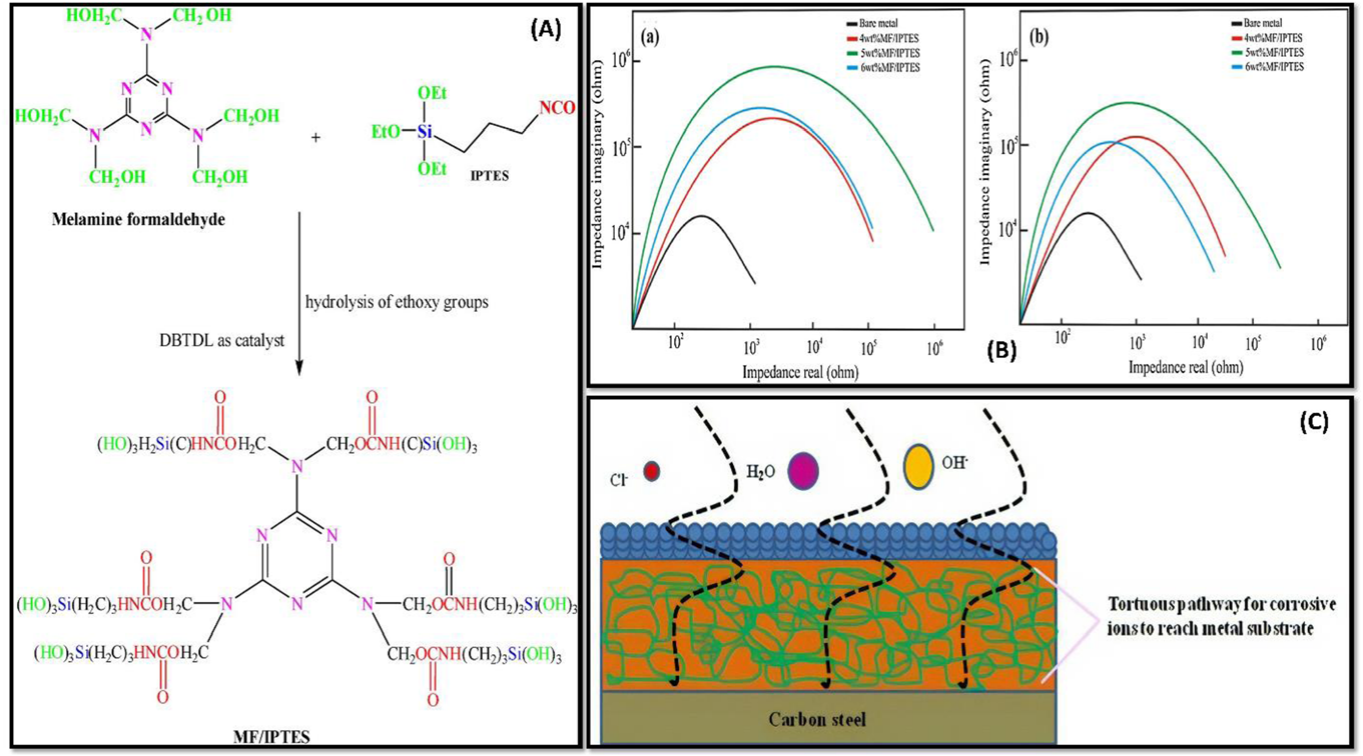
(A) Schematic diagram representing IPTES functionalization of MF. (B) Nyquist plot representation of WLA network in 3.5 wt% HCl (a) in 3.5 wt% NaOH (b) medium (C) digital illustration of the WLA coatings as torturous path for corrosive ions to reach metal substrate cured with MF/IPTES. This figure has been adapted from ref. 98 with permission from Elsevier, copyright 2018.

Due to its rich inherent properties, linseed oil is one of the highly utilised vegetable oils in the coating industry. Linseed oil has been widely used among researchers because it is eco-friendly, economical, available, and suitable to form the polymeric network.^79^ Drying oil Linseed oil-derived polymers are known for their self-healing abilities, and the recent advance in properties and applications for the same has been discussed in Table 4. Due to the drying ability of linseed oil has been used as a healing component within the microcapsules in self-healable polymeric coatings. However, temperature curing is one of the major challenges with the vegetable oil-based polymeric coating. Manawwer and coworkers has synthesized a linseed oil-based polyester-amide for anti-corrosion applications.^95^ The reported coating prepared was cured at the ambient environment, which possess appreciable anti-corrosion and physio-mechanical properties. Initially, linseed oil was used to form N, *N*-bis 2-hydroxy ethyl linseed oil fatty amide diol (HELA), followed by the formation of hydroxyl-terminated polyester-amide (Ed-PEA) by treating with ethylenediaminetetraacetic acid (EDTA). The resultant material was cured while reacting with poly (styrene co-maleic anhydride) (SMA) at ambient temperature. Polyester-amide coating was thermally stable upto 150 °C and possesses significant corrosion inhibiting properties. Subsequently, polyester amines have also been known for their uses in anti-bacterial and anti-corrosion coatings. To improve the anti-bacterial properties, durability and anti-corrosion properties, H. Abd El-Wahab and coworkers modified polyester amine with *N*-phthaloyl-glutamic acid.^96^ In this study, linseed oil and diethanolamine (DEA) reacted to get *N*,*N*-bis-(2-hydroxyethyl)linseed oil fatty acid amide (HELA) which was made to treat with phthalic anhydride (PA) and *N*-phthaloyl-glutamic acid (NPGA) (synthesised *in situ*) to obtain modified resin for coating. The reported coating exhibits notable chemical resistance and anti-corrosion properties along with excellent mechanical properties. The coating was tested to have less permeability for water, oxygen, and other ions to pass, owing to enriching the adhesion of the hydroxyl group connected to metal surfaces. Although bio oil-based nanocomposites are potential alternatives for replacing petroleum coatings, advancing the properties of composite polymers can do wonders to address the issues like corrosion, optical transparency, and flexibility. Shabnam Pathan and team attempted to address these noted issues by tailoring the linseed oil-based water-borne alkyd resin with 3-iso-cynato-propyl-tri-ethoxy silane (IPTES) and di-methyl-tin-di-neo-decanoate.^97^ In nanohybrid composite, flexibility is due to the synergistic reinforcing effect and merging of organic and inorganic phases of the system. Shabnam Pathan and coworkers used water-borne linseed oil-based alkyd composites to improve the mechanical properties,^98^ where silane-based coupling agents have been used to address the limitations and escalate the interaction between two phases. Cross-linked polymer coatings derived from linseed oil using melamine formaldehyde (MF) and 3 iso-cynato-propyl tri-ethoxy silane (IPTES) exhibits great mechanical and anticorrosive performance [Fig. 5A]. It was found that the prepared coating exhibits remarkable adhesive and barrier properties against chemical environment and water along with high thermal stability which comprises to corrosion inhibition [Fig. 5B and C].

**Table 4:** Linseed oil-based bio-polymer synthesis conditions, properties and applications

S. No.	Monomers	Resultant polymer	Catalyst/chain extender (conditions)	Polymerization technique/crosslinking	Fillers	Properties			References
						Self-healing	Mechanical properties	Anti-corrosion	
1	Linseed oil + poly (styrene co-maleic anhydride) (SMA)+ ethylene glycol monomethyl ether + sodium methoxide + ethylene diamine-tetra acetic acid	Polyester-amide	Ethylene-diaminetetraacetic acid (EDTA)	Cured at ambient environment	—	—	Thermal stability up to 150°, stretching hardness up to 3.7%, impact resistance-150 lb per inch, *T*_g_-120 °C	Anti-corrosion in 5% NaOH and HCl up tp 10 days	95
2	Linseed + diethanolamine (DEA)+ xylene + l-glutamic acid	Poly-ester-amide resins	—	Esterification	*N*-phthaloyl-glutamic acid(NPGA) ring	—	Impact resistance up to 1.4 kg m^−1^, excellent stretching of 1 kg, and flexibility	Anti-corrosion coating with acidic and alkaline resistance	96
3	Linseed oil+ 3-isocyanatopropyltriethoxysilane (IPTES)+ dimethyltin dineodecanoate + tetrahydro furan (THF)	Alkyd resin	3-Isocyanatopropyl-triethoxysilane (IPTES)	Sol–gel reaction	Organo-silane as nano-filler	—	Thermal stability-330 °C, *T*_g_ = 90–125 °C, contact angle = 97°, 2*Θ* = 20°	Anti-corrosion on CS with *E*_corr_ = −0.289 V, *I*_corr_ = 4.41 × 10−^10^ A cm^−2^, *R*_p_ = 1.05 × 10^8^ ohm (acid)& 1.94 × 10^5^ ohm(alkaline), corrosion rate-5.12 × 10^−6^ mpy	97
4	Linseed oil + melamine-formaldehyde (MF) + 3-iso-cynato-propyl tri-ethoxy silane (IPTES)+ phthalic acid	Water-borne alkyd resin	dibutyltin di laurate (DBTDL)+ p-toluene-sulfonic acid (PTSA) used as catalyst	Si-0-Si linkage	3-iso-Cynato-propyl tri-ethoxy silane (IPTES)	—	Adhesive properties, thermal stability, impact resistance, scratch hardness, 2*θ* = 20°	Anti-corrosion coating on carbon steel	98
5	Linseed oil + adipic acid + glycerol + CuSO4+ sodium hydroxide + ascorbic acid + manganese actuate + cobalt octoate + lead octoate	Hyperbranched alkyd resin	Dibutyltin oxide as catalyst	Polycondensation reaction	Cu_2_O nano-cubes as nano-filler	—	Thermal stability up to-325 °C, contact angle-120°, pull off test 26 MPa, impact resistance = 18 joule	Anti-corrosion inhibition on carbon steel with 500 hour spray resistance test in NaOH & HCl	79
6	Linseed oil+ 4-methoxypheno + triethylamine + para-toluene sulfonic acid + sodium hydroxide + itaconic acid	Epoxidized alkyd resin	Amberlite	Esterification	3-Amino propyl-tri-methoxy-silane (APTMS)	—	Thermal stability = 246 °C, *T*_g_ = 39–46 °C, stretch hardness-2250 g, scrub test>200 and static heat resistance-157 °C	Anti-corrosion properties with polarisation resistance = 0.089Ω, *E*_corr_ = 1131 mV, corrosion rate = 0.29917 mmpy, corrosion inhibition-94.02%	99
7	Linseed oil + polyvinyl alcohol (PVA)+ sodium dodecyl sulphate (SDS)+ diglycidyl ether of bisphenol A	Epoxy resin	3-Aminopropyl-trimethoxy silane (APS) as coupling agent	Sol–gel technique	Microcapsules filled with linseed oil	Self-healing properties	Tensile strength = 1.75 MPa, young's modulud-1515.4 MPa, elongation break-5.6%,stress break = 65.3 MPa	Anti-corrosion properties with corrosion resistance up to 7.94 × 10^5^ ohm cm^2^	101
8	Linseed oil + NaNO_3_ + resorcinol + polyvinyl alcohol (PVA)	Epoxy resin	NaNO_3_ used as corrosion inhibitor, linseed oil as healing agent	—	Talc nano-particles and urea-formaldehyde micro-capsules, sodium nitrate-NaNO_3_	Self-healing efficiency 99.9%	Thermal stability up to 350 °C, phase angle = −85 to-90°	Anti-corrosion properties with Rp = 0.01 mΩ, corrosion efficiency-99.5%	102
9	Linseed oil + tetra-*n*-butyl ammonium bromide (TBAB)+ diethylenetriamine (DETA)	Polyurethane	—	Epoxidation and carbonation	—	—	Contact angle = 75°, thermal stability-394 °C, pull off adhesion-5.1 MPa, direct impact = 80 in.lb	Anti-corrosion with impedence modulus = 1.85 × 10^6^ Ω cm^2^	104
10	Linseed oil + di-hemiacetal-ester (1,10-dibutoxyethyl-sebacate)+ *N*,*N*-dimethyl-acrylamide + dopamine hydrochloride	Acrylated-epoxidised resin	(3,4-Dihydroxyphenetyl) acrylamide as adhesion promoter	Copolymerization	1,10-Dibutoxyethyl-sebacate (DBES)	Self-healing properties	High hydrophobicity, *T*_g_ = −61 °C, thermal stability-200 °C	Anti-corrosion properties, barrier properties with *R*_f_ = −1.9 × 10^7^ Ω cm^2^, *R*_pore_ = 1.3 × 10^5^ Ω cm^2^	103
11	Linseed oil + aq poly (vinyl alcohol) (PVA)+ sodium lauryl sulphate (SLS)+ cardanol + methylene di phenyl diisocyanate(MDI)+ diethanolamine	Polyurethane	Resorcinol as cross linker, DBTDL as a catalyst	Condensation polymerisation	Microcapsules with 87% release core	Self-healing	Hydrophobic nature with contact angle-98.3°, cross cut adhesion = 100%, gloss% = 85%, thermal stability-230 °C	Anti-corrosion propertieswith corrosion inhibition efficiency = 86.92%	63

In another approach, Mohamed and coworkers introduced Cu_2_O nano-cube based filler into alkyd resin to achieve high thermal stability (∼324 °C) along with better mechanical and corrosion inhibition properties.^79^ The hyper-branched poly-alkyd resin was synthesized by adopting A_2_ + B_3_ approach, where glycerol and adipic acid were reacted in the presence of dibutyltin oxide (catalyst). The prepared materials were and further treated with linseed oil fatty acid and *P*-TSA as catalyst under poly-condensation reaction. The resultant resin incorporated with Cu_2_O nanoparticles was used for coatings, where coating possess excellent corrosion inhibition abilities, significant mechanical features as well as stability against chemicals. For further advancement of the study in vegetable oil-based alkyd resin Deepak M. Patil and coworkers synthesised epoxidized alkyd resin from linseed oil using itaconic acid for the application as anti-corrosion coating.^99^ It was a two-step process, where linseed oil was converted into mono-glyceride *via* an esterification process. Furthermore, the unsaturated binds present in the alkyd moity were converted into oxirane and later to epoxidized alkyd resin. The resultant polymer was modified by using 3-amino propyl-trimethoxy-silane (APTMS) to obtain a coating with good mechanical and adhesive properties. It is due to its ability to form H-bonding with the metal surface. The coating was able to create a good barrier against acid alkaline medium and corrosion and showed increased thermal stability up to 247 °C and high cross-linking density.

**Fig. 6:**
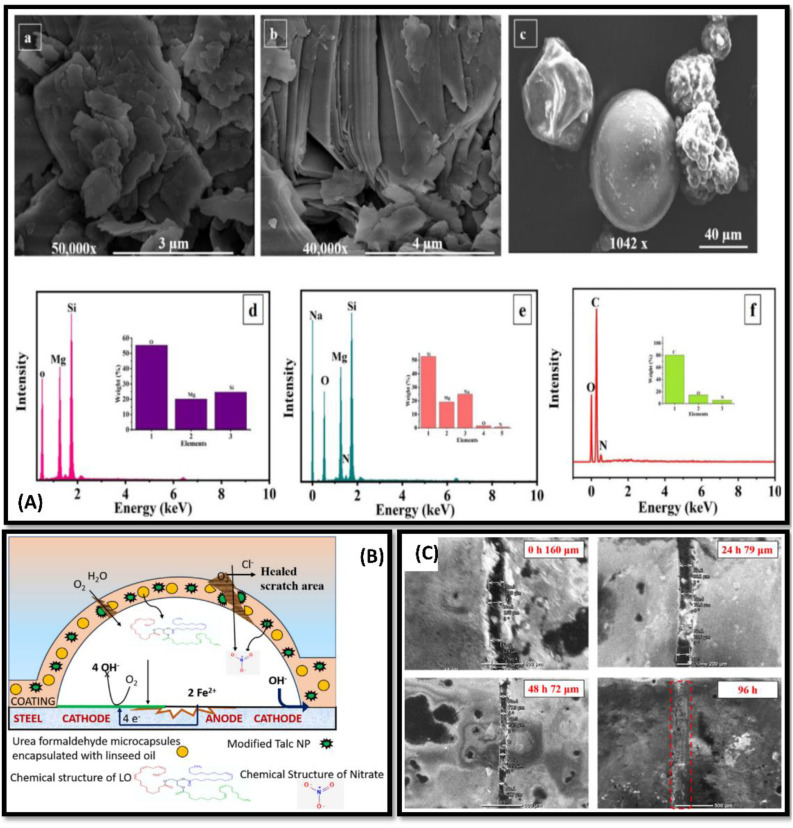
(A) FE-SEM and EDS analysis, of (a and d) as-received TNPs, (b and e) TNPs loaded with NaNO3 and (c and f) encapsulated UFMCs (B) schematic representation demonstrating the self-healing and corrosion inhibition mechanism of the developed polymeric nanocomposite coating (C) FE-SEM analysis of the polymeric nanocomposite coatings containing 3 wt% loaded TNPs at different intervals of time (0, 24, 48, 96 h). This figure has been adapted from ref. 70 with permission from Elsevier, copyright 2021.

Regarding microcapsule-based matrix, incompatibility of microcapsules with polymeric metric results in poor mechanical properties of the coating.^100^ To address these issues, Mirabedini and coworkers have modified the microcapsules with 3-aminopropyltrimethoxy silane coupling agent to improve the surface interaction.^101^ Microcapsules generated from polyurea-formaldehyde filled with linseed oil were found to exhibit self-healing properties. The reported polymeric matrix shows improved tensile strength and anti-corrosion properties. To create a coating with enhanced anti-corrosion and self-healing properties, Sehrish and coworkers made an attempt to incorporate of talc nanoparticles (TNP) and urea-formaldehyde micro (UFM) capsules[Fig. 6A].^102^ Talc falls into the category of mineral clay having corrosion inhibitor properties, composed of magnesium silicate [Fig. 6B]. The authors observed that composite exhibit excellent thermal stability and self-healing properties. When any damage occurs on the coating surface, it makes microcapsule breaks due to mechanical stress and linseed oil releases[Fig. 6C]. Linseed oil works as a healing agent as it starts to cross-link when it interacts with air and forms a protective film with 99% healing efficiency. In combination with NaNO_3_, this film inhibits corrosion from spreading on the substrate surface with a corrosion inhibition efficiency 99%. Likewise, to extend the lifetime of the oil-based polymeric coatings. David and coworkers used acrylated epoxidized linseed oil as the base matrix.^103^ Linseed oil was converted into acrylated epoxidized linseed oil and copolymerised with dihemiacetal ester (1,10-dibutoxyethyl sebacate (DBES)) and 3,4-dihydroxyphenetyl acrylamide(DHPA) for adding desired cross-linking property to the polymeric matrix. The corrosion protection by the coating on the surface was found to be appreciable, as DBES addition to the matrix results in an increase in hydrophobic property and high thermal stability, however, it does not lead the matrix towards barrier properties. Though interesting barrier properties were observed in absence of DB.

### 2.4. Other vegetable oils

Due to the difference in their functionality and degree of unsaturation present in their molecular structures, vegetable oils exhibit their unique features and qualities to deliver specific properties, as discussed in Table 5. In the coating network, drying time and durability of the matrix have quite an important roles, which varies oil to oil. Characteristics of neem oil are full of surprises as it delivers an enormous number of properties to the polymeric matrix. Neem oils great potential in the coating industry as it contains anti-corrosion and anti-microbial and is highly available (in India). Counting on it, Chaudhari and coworkers studied self-healing poly(ester-amide) synthesised from neem oil and their properties.^105^ Neem oil underwent amidation reaction with a diethanolamine and PbO as catalyst to result into Azadirachta indica juss Fatty-amide (AIJFA), which was used to synthesis polyester amide. Microcapsules were prepared using poly-amido-amine (PAMAM) and linseed oil as core material in it and further used for the development of polyurethane coatings. The prepared coating shows thermal stability up to 221 °C, resistance against water and chemical interaction including good adhesive and anti-corrosion properties on the metal substrate.

**Table 5:** Bio-polymer synthesis conditions, properties and applications generated from various types of oils

S. No.	Monomers	Resultant polymer	Catalyst/chain extender (conditions)	Polymerization technique/crosslinking	Fillers	Properties			References
						Self-healing	Mechanical properties	Anti-corrosion	
1	Neem oil + diethanolamine + poly-amido-amine (PAMAM) + methylene diphenyl diisocyanate(MDI) + toluene 2,4-ddisocyanates	Polyurethane	PbO as a catalyst	Amidation	Polyurea microcapsules filled with linseed oil	Self-healing with 4% microcapsules	Thermal stability up to 221 °C,cross cut adhesion-98, impact resistance50.90 lb per inch	Anti-corrosion coating	105
2	Mesua ferrea L. seeds + 2,4-toluene diisocyanate (TDI)+ glycerol and poly(e-caprolactone) diol (PCL) + *N*,*N*-dimethylformamide (DMF)	Hyper-branched polyurethane (HBPU)		Standard glycerolysis procedure	MWCNTs (diameter 10–20 nm and length 20 lm)	Shape recovery to the extent of 98.5%, shape fixity up to 92.2%	Melting temperature increased from 47 to 56 °C, non-toxicity at the cellular level and biodegradability, thermal stability up to 243 °C	—	111
2	Palm oil + phthalic anhydride (PA) + formaldehyde + lithium hydroxide(LiOH)	Epoxy resin	—	Polycondensation	Microscapsules filled with palm oil-based alkyd	Healing properties	Flexural strength-48 MPa, thermal stability-258 °C	—	112
3	Olive oil extract + urethane Pre-polymer + epoxy vinyl Ester + PDES + dimethyl aniline (DMA)	Epoxy and polyurethane	DBTL as catalyst, benzoyl peroxide (BPO) as initiator, chlorobenzene as stabilizer	—	Ethanol extracted olive leaf	Self-healing properties	Phase angle-80°	Anti-corrosion with *E*_corr_ = −721 mV, *I*_corr_ = 9 μA cm^2^, inhibition efficiency = 90.2%	113
4	Corn oil + isosorbide + iso-phorone di-iso-cyanate	poly(urethane-ether-amide)	Sulfuric acid + DBTDL	—	Fumed silica nanoparticles	—	Scratch resistance = 150lb/inch, bending ability = 1/8 inch, cross hatch test = 98%, gloss-72, thermal stability = 275 °C	Anti-corrosion properties on mild steelimpedence modulus-6.6 × 10^5^ Ω,inhibition efficiency = 99.7%	106
5	Jatropha oil + tri-methyl-ol-propane tri-acrylate(TMPTA)+ triethanolamine	Epoxy acrylate resin	1,4-Methoxyphenol and triethylamine as catalyst	—	Zinc oxide nano particles	—	Hydrophobic nature, barrier properties, scratch resistance = 0.7 kg, pendulum hardness-34.9%, gel hardness-93.4%	Anti-corrosion properties on mild steel	108
6	Tung oil + ammonium chloride + sodium chloride + poly-(ethylene-alt-maleic-anhydride)(EMA)+ Resorcinol + sodium dodecyl sulphate (SDS)	Alkyd resin	Zinc phosphate as corrosion inhibitor	—	Microcapsules	Self-healing property	60 days shelf life	Anti-corrosion properties with *R*_f_-844 KΩ, *R*_s_ = 178 mΩ, warburg impedence-0133 m Ω	109
7	Corn oil + isophorone diisocynate (IPDI)+ malonic acid + diethanolamine	poly(urethane-malonic-ester-amide)	—	Esterification, amidation	Carbon nano tubes	—	Hydrophobic with contact angle-110°, scratch hardness-2.7 kg, thermal stability- 322 °C, cross hatch-100%,impact resistance-150ln/inch	Anti-corrosion properties with corrosion potential = −0.593 V, *E*_corr_ = 1.166 × 10^−6^ mV, corrosion rate-1.35 × 10^−2^ mmpy	107
8	Tung oil + polyvinyl alcohol + resorcinol + ammonium chloride (NH4Cl) + hydrochloric acid (HCl)	Epoxy resin	—	—	Microcapsules	Self-healing properties	Thermal strength up tp 200 °C, adhesion strength-3.4 MPa,	Anti-corrosion protection with Rc-0.7 × 10^9^ Ω cm^2^. Corrosion resistance-10^6^ Ω cm^2^	110

To add on to green composite materials with improved properties, Manawwer and coworkers studied corn oil-based organic coating using isosorbide.^106^ Corn fatty amide reacted with isosorbide in the presence of sulfuric acid as catalyst to get poly(isosorbide-ether-amide) (PIEtA), which was further converted into PUIEtA/fused silica nanocomposite. Prepared silica based nanocomposite manifests excellent anticorrosion properties and barrier against chemicals with the notable thermal stability of 275 °C and increased adhesion up to 98%, impact resistance with corrosion inhibition efficiency 99%. In addition, authors have investigated corn oil-based poly(urethane-malonic-ester-amide) network to improve the properties of the matrix by incorporating multi-walled carbon nanotubes (MWCNTs) as filler.^107^*N*,*N*-bis(2-hydroxyethyl) corn oil fatty amide (HECA) was reacted with malonic acid in toluene to obtain malonic polyester-amide(MPEA) followed by the preparation of poly(urethane-malonic-ester-amide) using iso-phorone diisocyanate (IPDI) which finally get incorporated with MWCNTs. The integration of CNTs in polymeric network increases contact angle (110°) and provides hydrophobic nature to it, making it suitable for anti-corrosion coatings with excellent thermal stability and mechanical performance. Aung and coworkers suggested that ZnO can be used as a nano-filler for corrosion inhibition in polymeric coating.^108^ To synthesize the epoxy acrylate of jatropha oil (AEJO), epoxidized jatropha oil was made to react with triethylamine, 4-methoxyphenol and acrylic acid, which is further processed to prepare the composite by reacting AEJO with reactive diluent tetra-methyl-ol-propane tri-acrylate (TMPTA) and photo-initiator 2-hydroxy-2-methylpropiphenone, followed by the addition of ZnO as nano-filler in different compositions. These nano-hybrid resins possess properties to protect the metal surface from corrosion owing to the hydrophobic nature of ZnO. Incorporating a 5% weight% ZnO loading showed significant enhancement in corrosion resistance and coating performance. Tung oil is being used in polymeric coating industries for its positive impact as filler inside microcapsules. A study by Gonçalves and co-researchers reported that composites prepared from poly(urea-formaldehyde) positively impact corrosion inhibition and self-healing properties.^109^ Zinc phosphate is being used as a corrosion inhibitor as the hydroxyl group in the base network reacts with zinc phosphate and results in an even more stable zin oxide film, helping to protect the substrate from corrosion.

**Fig. 7:**
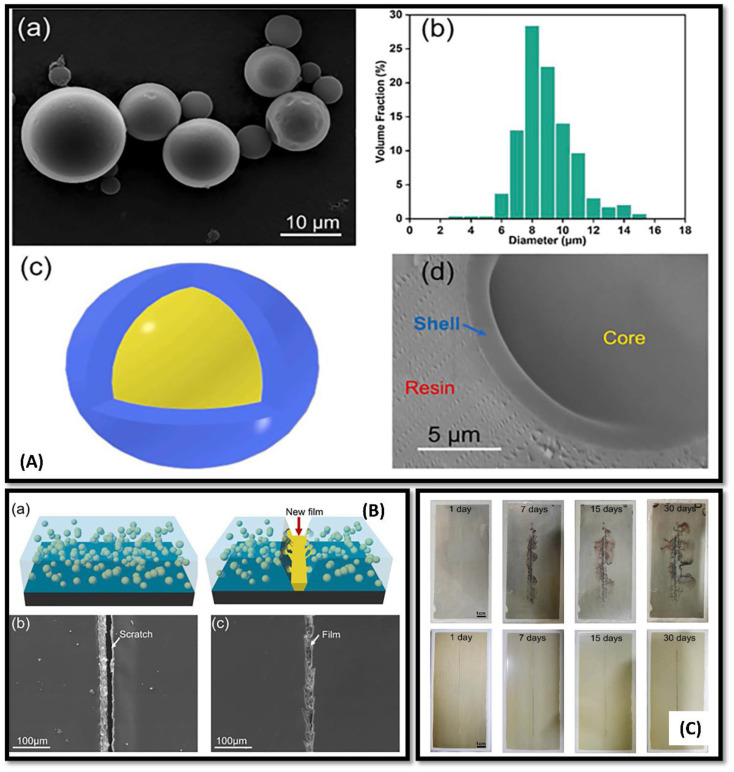
(A)(a) SEM image of prepared UF microcapsules, (b) size distribution study of the microcapsules prepared, (c) schematic diagram of core of shell structure, (d) SEM image representing thickness of prepared microcapsule shells (B) (a) schematic pictorial representation of the self-healing mechanism on scratched surface. (b) SEM image of the scratched control epoxy sample coating, (c) SEM image of the healed scratches on self-healing epoxy coated surface. (C) Optical image of AA2024-T3 plates protected from corrosion: AA2024-T3 plate coated with the control epoxy coating (up slides), AA2024-T3 plates coated with the self-healing material coatings (down slide) in salt spray test after 0, 7, 15, 30 days. This figure has been adapted from ref. 110 with permission from Elsevier, copyright 2021.

Along with all the properties of the coating, the thickness of the coating is also essential to study in polymeric coatings. Usually, in self-healing polymeric coatings, large microcapsules facilitate the release of a good amount of healing agents, which results in a thick coating. In comparison, a thin application of the coating is enough to protect the metal surface. Aiming toward the characteristic of diameter, Li and coworkers studied the tung oil microcapsules subsumed in epoxy matrix[Fig. 7A(a)].^110^ Tung oil-based microcapsules were prepared *in situ* using formaldehyde for the formation of shell and urea, resorcinol and ammonium chloride [Fig. 7A(c) and (d)]. Self-healing coating embedded with tung oil microcapsules was investigated, and found that a decrease in adhesion of coating was found, but the barrier ability of the coating was good along with self-healing and anti-corrosion properties [Fig. 7B and C].

## 3. Summary and future prospectives

In view of the governing procedures and pressing need to reduce CO_2_-emissions, the perspective of using biobased materials in the coating applications is attractive. The use of bio-chemical derived molecules mainly aims to reduce the number of synthetic molecules, however, achieving material performance similar to the commercial coating product represents a significant challenge. Certainly, in spite of great economic and ecological values, vegetable oil based polymeric coatings are facing numerous challenges in making place in industrial market,^114^*e.g.* polymeric network often possess low crosslinking density which lead towards lower in mechanical properties and solvent resistivity, due to the triglyceride long chain structure of vegetable oil based polymeric moiety, though it also offers flexibility to the network compared to the petroleum based polymeric networks.^5,20^ These drawbacks can be overcome through the addition of suitable crosslinker as well as by addition of fillers. In addition, to avoid the environmental oxidation and physical stability, vegetable oil based self-healing materials can be used in encapsulated form for developing self-healable polymeric coatings. But with encapsulated networks there are limitation in terms of reusability, re-processibility and low mechanical properties.^115,116^

As the low thermal stability of the VO based coating network ultimately lead towards the lower shelf life and hence the early degradation of the network, which can be answered by using the various fillers in the form of graphene based nanosheets, metal nanoparticles or metal-legend composites.^117,118^ On the other hand, vegetable oil based self-healing networks require particular conditions to reprocess and to reflect the characteristic behavior (like high temperature and high pressure), which is not feasible to provide at industrial level.^100,119^ To address such issues various techniques can be applied in case of advanced materials (*e.g.* vitrimers), where instead of conventional thermal heating electromagnetic radiation or induced heating can be used to attain the energy, required for the dynamic bond exchange reaction in order to facilitate the self-healing property at different working environment.^100,118^ To acquire the radiation heating of the system, the chemistry can be obtained with the presence of carbonaceous materials or the molecules with dielectric constant. In consideration of bio-integrated electronic devices, longer healing time of the cracks could result into failure of the device, apparently rapid healing of the network at ambient temperature would be required to avoid the damage.^120^ Overall, bio-molecules derived coatings can be advantageous when factors like the availability of the biobased additives and their true sustainability are considered. In addition, sometimes designing desirable molecules from petrochemicals is difficult, thus, availability of new bio-molecules/monomers opens the door for researchers to construct novel molecules difficult to be obtained from petrol-chemicals. Along with this, a bio-material design is made from scratch, allowing them to plan material properties in line with their future life cycle from the very beginning, thus, these functional materials could find versatile application by collaboration of chemist and industrial researchers.

## Conflicts of interest

There are no conflicts to declare.

## Supplementary Material

## References

[cit1] Olajire A. A., C. R. (2018). Recent advances on organic coating system technologies for corrosion protection of offshore metallic structures. J. Mol. Liq..

[cit2] Deka H., Karak N. (2009). Bio-based hyperbranched polyurethanes for surface coating applications. Prog. Org. Coat.

[cit3] Song Y. K., Kim H. W., Chung C. M. (2022). Repeatable Self-Healing of a Protective Coating Based on Vegetable-Oil-Loaded Microcapsules. Polymers.

[cit4] Uddin I. , Thomas S., Mishra R. K., and Asiri A. M., Sustainable polymer composites and nanocomposites, 2019, 10.1007/978-3-030-05399-4

[cit5] Nayak P. L. (2000). Natural oil-based polymers: Opportunities and challenges. J. Macromol. Sci., Polym. Rev..

[cit6] Krishnakumar B., Bose D., Singh M., Siva Prasanna Sanka R. V., Gurunadh V. V. S. S., Singhal S., Parthasarthy V., Guadagno L., Vijayan P P., Thomas S., Rana S. (2021). Sugarcane Bagasse-Derived Activated Carbon- (AC-) Epoxy Vitrimer Biocomposite: Thermomechanical and Self-Healing Performance. Int. J. Polym. Sci..

[cit7] Lamm M. E., Song L., Wang Z., Lamm B., Fu L., Tang C. (2019). A facile approach to thermomechanically enhanced fatty acid-containing bioplastics using metal-ligand coordination. Polym. Chem..

[cit8] Rana S., Solanki M., Sahoo N. G., Krishnakumar B. (2022). Bio-Vitrimers for Sustainable Circular Bio-Economy. Polymers.

[cit9] Chakraborty I., Chatterjee K. (2020). Polymers and Composites Derived from Castor Oil as Sustainable Materials and Degradable Biomaterials: Current Status and Emerging Trends. Biomacromolecules.

[cit10] Siyanbola T. O., Neelambaram P., Mohanty S., Somisetti V., Basak P., Narayan R., Kothapalli R. V. (2019). The effects of carbonized Eucalyptus globulus leaves on castor seed oil based urethane coating system. Prog. Org. Coat.

[cit11] Krishnakumar B., Sanka R. V. S. P., Binder W. H., Parthasarthy V., Rana S., Karak N. (2020). Vitrimers: Associative dynamic covalent adaptive networks in thermoset polymers. Chem. Eng. J..

[cit12] Islam M. R., Beg M. D. H., Jamari S. S. (2014). Development of vegetable-oil-based polymers. J. Appl. Polym. Sci..

[cit13] Liang H. (2018). *et al.*
, Bio-based cationic waterborne polyurethanes dispersions prepared from different vegetable oils. Ind. Crops Prod..

[cit14] Akbar E., Yaakob Z., Kamarudin S. K., Ismail M., Salimon J. (2009). Characteristic and composition of Jatropha curcas oil seed from Malaysia and its potential as biodiesel feedstock feedstock. Eur. J. Appl. Eng. Sci. Res..

[cit15] Galúcio C. S., Souza R. A., Stahl M. A., Sbaite P., Benites C. I., Maciel M. R. W. (2011). Physicochemical characterization of monoacylglycerols from sunflower oil. Procedia Food Sci..

[cit16] Abdalla I. I. H., Boussab a, El Garrouj D., Souhial B. (2014). Physical and Chemical Characteristics of Olive Oils from Cooperatives for Olive Growers in the North of Morocco. Int. J. Basic Appl. Sci..

[cit17] Azcan N., Danisman A. (2007). Alkali catalyzed transesterification of cottonseed oil by microwave irradiation. Fuel.

[cit18] Ramanujam S., Zequine C., Bhoyate S., Neria B., Kahol P., Gupta R. (2019). Novel Biobased Polyol Using Corn Oil for Highly Flame-Retardant Polyurethane Foams. C.

[cit19] Silva J. A. C., Grilo L. M., Gandini A., Lacerda T. M. (2021). The prospering of macromolecular materials based on plant oils within the blooming field of polymers from renewable resources. Polymers.

[cit20] Stanzione J., La Scala J. (2016). Sustainable polymers and polymer science: Dedicated to the life and work of Richard P. Wool. J. Appl. Polym. Sci..

[cit21] Loesch-Zhang A., Cordt C., Geissler A., Biesalski M. (2022). A Solvent-Free Approach to Crosslinked Hydrophobic Polymeric Coatings on Paper Using Vegetable Oil. Polymers.

[cit22] Pettazzoni L., Leonelli F., Martinelli A., Migneco L. M., Alfano S., Luca D. D., Celio L., Lisio V. D. (2022). Transamidation-based vitrimers from renewable sources. J. Appl. Polym. Sci..

[cit23] Rana S., Karak N., Cho J. W., Kim Y. H. (2008). Enhanced dispersion of carbon nanotubes in hyperbranched polyurethane and properties of nanocomposites. Nanotechnology.

[cit24] Altuna F. I., Antonacci J., Arenas G. F., Pettarin V., Hoppe C. E., Williams R. J. J. (2016). Photothermal triggering of self-healing processes applied to the reparation of bio-based polymer networks. Mater. Res. Express.

[cit25] Zhang C. (2021). *et al.*
, Renewable Castor-Oil-based Waterborne Polyurethane Networks: Simultaneously Showing High Strength, Self-Healing, Processability and Tunable Multishape Memory. Angew. Chem., Int. Ed..

[cit26] Binder W. H. , Self-healing polymers: from principles to applications, John Wiley & Sons, 2013, 10.1002/9783527670185

[cit27] Cho S. H., White S. R., V Braun P. (2009). Self-healing polymer coatings. Adv. Mater..

[cit28] Long R., Qi H. J., Dunn M. L. (2013). Modeling the mechanics of covalently adaptable polymer networks with temperature-dependent bond exchange reactions. Soft Matter.

[cit29] Zhang Y. (2016). *et al.*
, Malleable and Recyclable Poly(urea-urethane) Thermosets bearing Hindered Urea Bonds. Adv. Mater..

[cit30] Guadagno L. (Jan. 2019). *et al.*
, Self-healing epoxy nanocomposites via reversible hydrogen bonding. Composites, Part B.

[cit31] Krishnakumar B., Pucci A., Wadgaonkar P. P., Kumar I., Binder W. H., Rana S. (2022). Vitrimers based on bio-derived chemicals: Overview and future prospects. Chem. Eng. J..

[cit32] Sanka R. V. S. P., Krishnakumar B., Leterrier Y., Pandey S., Rana S., Michaud V. (2019). Soft self-healing nanocomposites. Front. Mater..

[cit33] Hong Y., Su M. (2012). Multifunctional self-healing and self-reporting polymer composite with integrated conductive microwire networks. ACS Appl. Mater. Interfaces.

[cit34] Hu M. (2018). *et al.*
, Monitoring crack appearance and healing in coatings with damage self-reporting nanocapsules. Mater. Horiz..

[cit35] Guadagno L. (2019). *et al.*
, Reversible self-healing carbon-based nanocomposites for structural applications. Polymers.

[cit36] Campanella A., Döhler D., Binder W. H. (2018). Self-Healing in Supramolecular Polymers. Macromol. Rapid Commun..

[cit37] Thakur V. K., Kessler M. R. (2015). Self-healing polymer nanocomposite materials: A review. Polymer.

[cit38] Fischer H. (2010). Self-repairing material systems–a dream or a reality?. Nat. Sci..

[cit39] Lodish H. , Berk A., Matsudaira P., Kaiser C. A., Krieger M. and Scott M. P., Molecular Cell Biology, W. H. Freeman, 5th edn, 2003

[cit40] Montarnal D., Capelot M., Tournilhac F., Leibler L. (2011). Silica-like malleable materials from permanent organic networks. Sci..

[cit41] Denissen W., Winne J. M., Du Prez F. E. (2016). Vitrimers: Permanent organic networks with glass-like fluidity. Chem. Sci..

[cit42] Yue C. (2022). *et al.*
, Vitrimeric silicone composite with high thermal conductivity and high repairing efficiency as thermal interface materials. J. Colloid Interface Sci..

[cit43] Wang M. (2022). *et al.*
, Rapid self-healed vitrimers via tailored hydroxyl esters and disulfide bonds. Polymer.

[cit44] Wang H., Guo S., Zhang X., Liu Y., Liu T., Yu H. (2022). Materials & Design Insight into the structure-property relationships of intramolecularly- catalyzed epoxy vitrimers. Mater. Des..

[cit45] Jia P. (2022). *et al.*
, Bio-based and degradable vitrimer-graphene/graphene oxide composites with self-healing ability stimulated by heat, electricity and microwave as temperature and fire warning sensors. Compos. Sci. Technol..

[cit46] Nardeli J. V., Fugivara C. S., Taryba M., Montemor M. F., Ribeiro S. J. L., Benedetti A. V. (2020). Novel healing coatings based on natural-derived polyurethane modified with tannins for corrosion protection of AA2024-T3. Corros. Sci..

[cit47] Yang X., Guo L., Xu X., Shang S., Liu H. (2020). A fully bio-based epoxy vitrimer: Self-healing, triple-shape memory and reprocessing triggered by dynamic covalent bond exchange. Mater. Des..

[cit48] Song Y.-K., Kim H.-W., Chung C.-M. (2022). Repeatable Self-Healing of a Protective Coating Based on Vegetable-Oil-Loaded Microcapsules. Polymers.

[cit49] Rana S., Döhler D., Nia A. S., Nasir M., Beiner M., Binder W. H. (2016). ‘Click’ -Triggered Self-Healing Graphene Nanocomposites. Macromol. Rapid Commun..

[cit50] Yuan L., Zhang Y., Wang Z., Han Y., Tang C. (2019). Plant Oil and Lignin-Derived Elastomers via Thermal Azide-Alkyne Cycloaddition Click Chemistry. ACS Sustainable Chem. Eng..

[cit51] Nia A. S., Rana S., Döhler D., Noirfalise X., Belfiore A., Binder W. H. (2014). Click chemistry promoted by graphene supported copper nanomaterials. Chem. Commun..

[cit52] Keshmiri N., Najmi P., Ramezanzadeh B., Bahlakeh G. (2021). Superior thermal-mechanical properties of the epoxy composite reinforced with rGO-ATMP; Combined DFT-D theoretical modeling/experimental studies. J. Mol. Liq..

[cit53] Najmi P., Keshmiri N., Ramezanzadeh M., Ramezanzadeh B. (2021). Highly improving the mechanical-responses/thermal-stability of the epoxy nano-composite using novel highly-oxidized multi-walled carbon nanotubes (OMWCNT) functionalized by Zinc-doped Polyaniline (PANI) nanofibers. J. Taiwan Inst. Chem. Eng..

[cit54] Keshmiri N., Najmi P., Ramezanzadeh M., Ramezanzadeh B. (2021). Designing an eco-friendly lanthanide-based metal organic framework (MOF) assembled graphene-oxide with superior active anti-corrosion performance in epoxy composite. J. Cleaner Prod..

[cit55] Keshmiri N., Najmi P., Ramezanzadeh B., Ramezanzadeh M., Bahlakeh G. (2021). Nano-scale P, Zn-codoped reduced-graphene oxide incorporated epoxy composite; synthesis, electronic-level DFT-D modeling, and anti-corrosion properties. Prog. Org. Coat.

[cit56] Keshmiri N., Najmi P., Ramezanzadeh M., Ramezanzadeh B., Bahlakeh G. (2022). Ultrastable Porous Covalent Organic Framework Assembled Carbon Nanotube as a Novel Nanocontainer for Anti-Corrosion Coatings: Experimental and Computational Studies. ACS Appl. Mater. Interfaces.

[cit57] Guadagno L. (2019). *et al.*
, Functional structural nanocomposites with integrated self-healing ability. Mater. Today: Proc..

[cit58] Haddadi S. A. (2022). *et al.*
, Cerium-doped tannic acid-reduced graphene oxide nanoplatform/epoxy nanocomposite coatings with enhanced mechanical and Bi-functional corrosion protection properties. Composites, Part B.

[cit59] Patil C. K., Jung D. W., Jirimali H. D., Baik J. H., Gite V. V., Hong S. C. (2021). Nonedible vegetable oil-based polyols in anticorrosive and antimicrobial polyurethane coatings. Polymers.

[cit60] Akram D., Sharmin E., Ahmad S. (2008). Synthesis, characterization and corrosion protective properties of boron-modified polyurethane from natural polyol. Prog. Org. Coat.

[cit61] Sharmin E., Zafar F., Akram D., Alam M., Ahmad S. (2015). Recent advances in vegetable oils based environment friendly coatings: A review. Ind. Crops Prod..

[cit62] Asif A. H. (2022). *et al.*
, Enhancement of Anticorrosive Performance of Cardanol Based Polyurethane Coatings by Incorporating Magnetic Hydroxyapatite Nanoparticles. Materials (Basel).

[cit63] Mahajan M. S., Gite V. V. (2022). Self-healing polyurethane coatings of eugenol-based polyol incorporated with linseed oil encapsulated cardanol-formaldehyde microcapsules: A sustainable approach. Prog. Org. Coat.

[cit64] Dutta G. K., Karak N. (2022). Bio-based waterborne polyester/cellulose nanofiber-reduced graphene oxide–zinc oxide nanocomposite: an approach towards sustainable mechanically robust anticorrosive coating. Cellulose.

[cit65] Mahapatra S. S., Karak N. (2004). Synthesis and characterization of polyesteramide resins from Nahar seed oil for surface coating applications. Prog. Org. Coat.

[cit66] Kar A., Karak N. (2022). Bio-based poly(ester amide): mechanical, thermal and biodegradable behaviors. J. Polym. Res..

[cit67] Morales-Cerrada R., Tavernier R., Caillol S. (2021). Fully bio-based thermosetting polyurethanes from bio-based polyols and isocyanates. Polymers.

[cit68] Zhang W. (2019). *et al.*
, High bio-content castor oil based waterborne polyurethane/sodium lignosulfonate composites for environmental friendly UV absorption application. Ind. Crops Prod..

[cit69] Thakur S., Barua S., Karak N. (2015). Self-healable castor oil based tough smart hyperbranched polyurethane nanocomposite with antimicrobial attributes. RSC Adv..

[cit70] Thakur S., Karak N. (2015). A tough, smart elastomeric bio-based hyperbranched polyurethane nanocomposite. New J. Chem..

[cit71] Shirke A., Dholakiya B., Kuperkar K. (2015). Novel applications of castor oil based polyurethanes: a short review. Polym. Sci., Ser. B.

[cit72] Bhat S. I., Ahmad S. (2018). Castor oil-TiO_2_ hyperbranched poly (ester amide) nanocomposite: a sustainable, green precursor-based anticorrosive nanocomposite coatings. Prog. Org. Coat.

[cit73] Kashif M., Anjum A. (2020). Chemical and electrochemical corrosion studies of ricinus communis oil based poly(urethane-ricinoleamide) coatings. J. Phys.: Conf. Ser..

[cit74] Patil A. M., Jagtap R. N. (2021). PU-coating performance of bio-based hyperbranched alkyd resin on mild steel and wood substrate. J. Coat. Technol. Res..

[cit75] Wei D., Zeng J., Yong Q. (2021). High-Performance Bio-Based Polyurethane Antismudge Coatings Using Castor Oil-Based Hyperbranched Polyol as Superior Cross-Linkers. ACS Appl. Polym. Mater..

[cit76] Lu J. (2021). *et al.*
, Self-healable castor oil-based waterborne polyurethane/MXene film with outstanding electromagnetic interference shielding effectiveness and excellent shape memory performance. J. Colloid Interface Sci..

[cit77] Thakur S., Karak N. (2013). Castor oil-based hyperbranched polyurethanes as advanced surface coating materials. Prog. Org. Coat.

[cit78] Karami Z., Zohuriaan-Mehr M. J., Kabiri K., Ghasemi Rad N. (2019). Bio-based thermoset alloys from epoxy acrylate, sesame oil- and castor oil-derived resins: Renewable alternatives to vinyl ester and unsaturated polyester resins. Polym. Renewable Resour..

[cit79] Selim M. S. and Shenashen M. A., Coatings for surface applications, 2018, pp. 10048–10058, 10.1039/c7nj03440g

[cit80] Siyanbola T. O. (2021). *et al.*
, Specific crosslinking effects of poly(epichlorohydrin)-triol on urethane polymer matrix of castor seed oil-based coatings. J. Coat. Technol. Res..

[cit81] Srisaard S., Amornkitbamrung L., Charoensuk K., Sapcharoenkun C., Jubsilp C., Rimdusit S. (2021). Effects of graphene nanoplatelets on bio-based shape memory polymers from benzoxazine/epoxy copolymers actuated by near-infrared light. J. Intell. Mater. Syst. Struct..

[cit82] Kocaman S., Karaman M., Gursoy M., Ahmetli G. (2017). Chemical and plasma surface modification of lignocellulose coconut waste for the preparation of advanced biobased composite materials. Carbohydr. Polym..

[cit83] Alagi P. (2018). *et al.*
, Functional soybean oil-based polyols as sustainable feedstocks for polyurethane coatings. Ind. Crops Prod..

[cit84] Song F. (2019). *et al.*
, Tunable ‘soft and stiff’, self-healing, recyclable, thermadapt shape memory biomass polymers based on multiple hydrogen bonds and dynamic imine bonds. J. Mater. Chem. A.

[cit85] Liu Y. Y., He J., Li Y. D., Zhao X. L., Zeng J. B. (2020). Biobased, reprocessable and weldable epoxy vitrimers from epoxidized soybean oil. Ind. Crops Prod..

[cit86] Aly K. I., Sun J., Kuckling D., Younis O. (2020). Polyester resins based on soybean oil: synthesis and characterization. J. Polym. Res..

[cit87] Capiel G., Hernández E., Marcovich N. E., Mosiewicki M. A. (2020). Stress relaxation behavior of weldable crosslinked polymers based on methacrylated oleic and lauric acids. Eur. Polym. J..

[cit88] Abdollahi Baghban S., Ebrahimi M., Khorasani M., Bagheri-Khoulenjani S. (2021). Self-stratifying behavior of a novel light-curable coating with gradient hydrophobic properties: Computational and experimental study. Prog. Org. Coat.

[cit89] Alagi P., Choi Y. J., Seog J., Hong S. C. (2016). Efficient and quantitative chemical transformation of vegetable oils to polyols through a thiol-ene reaction for thermoplastic polyurethanes. Ind. Crops Prod..

[cit90] Chen J., Liu H., Zhang W., Lv L., Liu Z. (2020). Thermosets resins prepared from soybean oil and lignin derivatives with high biocontent, superior thermal properties, and biodegradability. J. Appl. Polym. Sci..

[cit91] Özşeker A., Karadeniz K., Şen M. Y. (2019). Silylation of epoxidized soybean oil with triethoxysilanes, synthesis and characterization of their polyurethanes. Turk. J. Chem..

[cit92] Hu S., Chen X., Torkelson J. M. (2019). Biobased Reprocessable Polyhydroxyurethane Networks: Full Recovery of Crosslink Density with Three Concurrent Dynamic Chemistries. ACS Sustainable Chem. Eng..

[cit93] Hernández E., Mosiewicki M. A., Marcovich N. E. (2020). Bio-Based Polymers Obtained from Modified Fatty Acids and Soybean Oil with Tailorable Physical and Mechanical Performance. Eur. J. Lipid Sci. Technol..

[cit94] Ataei S., Hassan A., Yahya R. (2021). Dual Microcapsulation of an Environmentally-Friendly-Based Reactive Multifunctional Acrylated Epoxy Resin and Thiol by Internal Phase Separation Technique for Self-healing Applications. J. Polym. Environ..

[cit95] Alam M., Alandis N. M. (2011). Development of Ambient Cured Polyesteramide Coatings from Linseed Oil: A Sustainable Resource. J. Polym. Environ..

[cit96] Abd El-Wahab H., Abd El-Hai F., Naser A. M., El-Bialy Z. I., Mostafa M., Lin L. (2012). Synthesis and characterisation of new modified anti-corrosive polyesteramide resins by partial replacement of the ingredient source of the polybasic acid for organic surface coatings. Pigm. Resin Technol..

[cit97] Pathan S., Ahmad S. (2016). Synergistic Effects of Linseed Oil Based Waterborne Alkyd and 3-Isocynatopropyl Triethoxysilane: Highly Transparent, Mechanically Robust, Thermally Stable, Hydrophobic, Anticorrosive Coatings. ACS Sustainable Chem. Eng..

[cit98] Pathan S., Ahmad S. (2018). Progress in Organic Coatings Green and sustainable anticorrosive coating derived from waterborne linseed alkyd using organic-inorganic hybrid cross linker. Prog. Org. Coat.

[cit99] Patil D. M., Phalak G. A., Mhaske S. T. (2018). Design and synthesis of bio-based epoxidized alkyd resin for anti-corrosive coating application. Iran. Polym. J.

[cit100] Bei Y. (2022). *et al.*
, Recent progress of biomass based self-healing polymers. J. Appl. Polym. Sci..

[cit101] Mirabedini S. M., Farnood R. R., Esfandeh M., Zareanshahraki F., Rajabi P. (2020). Nanocomposite coatings comprising APS-treated linseed oil-embedded polyurea-formaldehyde microcapsules and nanoclay, part 2: Self-healing and corrosion resistance properties. Prog. Org. Coat.

[cit102] Habib S., Fayyed E., Abdul R., Kahraman R. (2021). Improved self-healing performance of polymeric nanocomposites reinforced with talc nanoparticles ( TNPs ) and urea-formaldehyde microcapsules (UFMCs). Arabian J. Chem..

[cit103] Nadine P., Boucher D., Ladmiral V., Negrell C., Causs N. (2021). Prog. Org. Coat.

[cit104] Pouladi J., Mirabedini S. M., Mohammadloo H. E., Rad N. G. (2021). Synthesis of novel plant oil-based isocyanate-free urethane coatings and study of their anti-corrosion properties. Eur. Polym. J..

[cit105] Chaudhari A. B., Tatiya P. D., Hedaoo R. K., Kulkarni R. D., Gite V. V. (2013). Polyurethane prepared from neem oil polyesteramides for self-healing anticorrosive coatings. Ind. Eng. Chem. Res..

[cit106] Alam M. (2019). Corn oil based poly(urethane-ether-amide)/fumed silica nanocomposite coatings for anticorrosion application. Int. J. Polym. Anal. Charact..

[cit107] Alam M., Alandis N. M., Alam J., Ahmad N., Alam M. A. (2021). Development of a poly(urethane-malonic-esteramide) coating from corn oil and carbon nanotubes for corrosion resistant applications. Int. J. Polym. Anal. Charact..

[cit108] Aung M. M. , Li W. J., and Lim H. N., “Improvement of Anticorrosion Coating Properties in Bio-Based Polymer Epoxy Acrylate Incorporated with Nano Zinc Oxide Particles,” 2020, 10.1021/acs.iecr.9b05639

[cit109] Cordeiro Neto A. G., Pellanda A. C., de Carvalho Jorge A. R., Floriano J. B., Coelho Berton M. A. (2020). Preparation and evaluation of corrosion resistance of a self-healing alkyd coating based on microcapsules containing Tung oil. Prog. Org. Coat.

[cit110] Li J., Shi H., Liu F., Han E. H. (2021). Self-healing epoxy coating based on tung oil-containing microcapsules for corrosion protection. Prog. Org. Coat.

[cit111] Yang Z., Peng H., Wang W., Liu T. (2010). Crystallization behavior of poly(ε-caprolactone)/layered double hydroxide nanocomposites. J. Appl. Polym. Sci..

[cit112] Shahabudin N., Yahya R., Gan S. N. (2016). Microcapsules filled with a palm oil-based alkyd as healing agent for epoxy matrix. Polymers.

[cit113] Jamshidnejad Z., Afshar A., RazmjooKhollari M. A. (2018). Synthesis of self-healing smart epoxy and polyurethane coating by encapsulation of olive leaf extract as corrosion inhibitor. Int. J. Electrochem. Sci..

[cit114] Adekunle K. F. (2015). A Review of Vegetable Oil-Based Polymers: Synthesis and Applications. Open J. Polym. Chem..

[cit115] Lammari N., Louaer O., Meniai A. H., Fessi H., Elaissari A. (2021). Plant oils: From chemical composition to encapsulated form use. Int. J. Pharm..

[cit116] Llevot A. (2017). Sustainable Synthetic Approaches for the Preparation of Plant Oil-Based Thermosets. J. Am. Oil Chem. Soc..

[cit117] Zhang Y., Hu J. (2020). Isocyanate Modified GO Shape-Memory Polyurethane Composite. Polymers.

[cit118] Song T. (2021). *et al.*
, Self-healing Materials: A Review of Recent Developments. ES Mater. Manuf..

[cit119] Fainleib A. M., Purikova O. H. (2019). Self-healing polymers: approaches of healing and their application. Polym. J..

[cit120] Xu J. (2022). *et al.*
, Advances and Challenges of Self-Healing Elastomers: A Mini Review. Materials.

